# Identification of Novel Candidate Epitopes on SARS-CoV-2 Proteins for South America: A Review of HLA Frequencies by Country

**DOI:** 10.3389/fimmu.2020.02008

**Published:** 2020-09-03

**Authors:** David Requena, Aldhair Médico, Ruy D. Chacón, Manuel Ramírez, Obert Marín-Sánchez

**Affiliations:** ^1^Laboratory of Cellular Biophysics, The Rockefeller University, New York, NY, United States; ^2^Laboratorio de Bioinformática, Biología Molecular y Desarrollos Tecnológicos, Laboratorios de Investigación y Desarrollo, Facultad de Ciencias y Filosofía, Universidad Peruana Cayetano Heredia, Lima, Peru; ^3^Departamento de Patologia, Faculdade de Medicina Veterinária e Zootecnia, Programa Interunidades em Biotecnologia, Universidade de São Paulo, São Paulo, Brazil; ^4^Unidad de Bioinformática, Centro de Investigaciones Tecnológicas, Biomédicas y Medioambientales, Lima, Peru; ^5^Departamento Académico de Microbiología Médica, Facultad de Medicina, Universidad Nacional Mayor de San Marcos, Lima, Peru

**Keywords:** allele frequency, HLA, literature review, South America, epitope, immunoinformatics, SARS-CoV-2, COVID-19

## Abstract

Coronavirus disease (COVID-19), caused by the virus SARS-CoV-2, is already responsible for more than 4.3 million confirmed cases and 295,000 deaths worldwide as of May 15, 2020. Ongoing efforts to control the pandemic include the development of peptide-based vaccines and diagnostic tests. In these approaches, HLA allelic diversity plays a crucial role. Despite its importance, current knowledge of HLA allele frequencies in South America is very limited. In this study, we have performed a literature review of datasets reporting HLA frequencies of South American populations, available in scientific literature and/or in the Allele Frequency Net Database. This allowed us to enrich the current scenario with more than 12.8 million data points. As a result, we are presenting updated HLA allelic frequencies based on country, including 91 alleles that were previously thought to have frequencies either under 5% or of an unknown value. Using alleles with an updated frequency of at least ≥5% in any South American country, we predicted epitopes in SARS-CoV-2 proteins using NetMHCpan (I and II) and MHC flurry. Then, the best predicted epitopes (class-I and -II) were selected based on their binding to South American alleles (Coverage Score). Class II predicted epitopes were also filtered based on their three-dimensional exposure. We obtained 14 class-I and four class-II candidate epitopes with experimental evidence (reported in the Immune Epitope Database and Analysis Resource), having good coverage scores for South America. Additionally, we are presenting 13 HLA-I and 30 HLA-II novel candidate epitopes without experimental evidence, including 16 class-II candidates in highly exposed conserved areas of the NTD and RBD regions of the Spike protein. These novel candidates have even better coverage scores for South America than those with experimental evidence. Finally, we show that recent similar studies presenting candidate epitopes also predicted some of our candidates but discarded them in the selection process, resulting in candidates with suboptimal coverage for South America. In conclusion, the candidate epitopes presented provide valuable information for the development of epitope-based strategies against SARS-CoV-2, such as peptide vaccines and diagnostic tests. Additionally, the updated HLA allelic frequencies provide a better representation of South America and may impact different immunogenetic studies.

## Introduction

The novel virus Severe Acute Respiratory Syndrome Coronavirus 2 (SARS-CoV-2) (1, 2) is the first member of the Betacoronavirus genus to reach pandemic status (3). This virus probably originated in bats and infects humans with the participation of an intermediate host (4–7), like its two highly human-infective relatives, SARS-CoV (8, 9) and MERS-CoV (10). It causes Coronavirus Disease 2019 (COVID-19), whose clinical symptoms include fever, cough, fatigue, sputum production, and difficulty breathing (11, 12). Transmission is mostly human-to-human through respiratory droplets and direct contact, carrying infectious virions to the nose, mouth, and eyes (13, 14). It has spread to 216 countries, resulting in more than 4.3 million confirmed cases and 295,000 deaths worldwide as of May 15, 2020 (15). It has an estimated basic reproductive number (*R*_0_) of 2.24–3.58 (16).

SARS-CoV-2, as with other Coronaviruses, is characterized by a high recombination frequency and mutation rate, in addition to a relatively large and sophisticated genetic machinery compared to other RNA viruses. At the 5′ end of the genome, the cleavable polyprotein ab (ORF1ab) is processed into 16 non-structural proteins (NSPs). They are involved in the viral replication and assembly process, as well as in immune evasion (17, 18). At the 3′ end, the structural proteins Spike (S), envelope (E), membrane (M), and nucleocapsid (N) are interspersed by the accessory proteins ORF 3a, 6, 7a, 7b, 8, and 10 (19, 20). A key factor in viral attachment and entry is the receptor-binding domain (RBD), located in the subunit 1 (S1) of the S protein. This binds strongly to the angiotensin-converting enzyme 2 (ACE2) receptors (21, 22). Other possible receptors, like CD209L (23), CD147 (24), and the protease TMPRSS2 (25), could also participate in the viral entry and processing.

Post-translational modifications (PTMs) are covalent modifications that regulate protein functions. In coronaviruses, they are required for a successful viral cycle. Glycosylation and palmitoylation of S and E proteins are fundamental in terms of stability, enzymatic activity, subcellular localization, and protein interaction (26–29). Similarly, glycosylation of the M protein (30, 31), phosphorylation, and ribosylation of the N protein (32, 33), as well as other PTMs in non-structural and accessory proteins, can play a determinant role in the viral cycle (34, 35). Considering the relevant role of PTMs and the complex composition of N-glycans, it was proposed that inhibition with N-butyl-deoxynojirimycin (NB-DNJ) (35) or the addition of carbohydrate-binding agents (CBAs) could be considered as therapeutic strategies against SARS-CoV infections (36). Nitric Oxide (NO) and its derivatives have been shown to inhibit SARS-CoV replication by reducing the palmitoylation on the nascent Spike protein, affecting the receptor binding. It also affects viral RNA production in the early steps of replication, potentially due to an effect on the ORF1a-encoded cysteine proteases (37).

Effective methods to control the pandemic include the development of vaccines and diagnostic tests. The fast release of complete SARS-CoV-2 genomes boosted the development of molecular diagnostic methods, resulting in an increasing portfolio of nucleic acid approaches like RT-qPCR (38–40), serological-based approaches like ELISA (41, 42), immunochromatographic panels based on antibodies IgM/IgG (43) or antigens (44), and hybrid systems in Point-of-Care devices, consisting of viral genome pre-amplification followed by a cleavage assay in a lateral flow system (45).

Vaccine development efforts are undergoing worldwide. There are 110 prophylactic vaccine candidates as of May 15, 2020. Three of them are based on live-attenuated virus, seven on inactivated virus, 27 on viral vectors (12 replicating and 15 non-replicating), 26 on nucleic acids (10 using DNA and 16 RNA), 38 on recombinant proteins, six on Virus-Like Particles (VLP), and three unknown (46, 47). Some vaccines have already moved to a clinical phase. There are six in Phase I: Pathogen-specific aAPC (NCT04299724, Shenzhen Geno-Immune Medical Institute, China), Recombinant Novel Coronavirus Vaccine (Adenovirus Type 5 Vector) (NCT04313127, CanSino Biologics Inc.), bacTRL-Spike (NCT04334980, Symvivo Corporation, Canada), INO-4800 (NCT04336410, Inovio Pharmaceuticals, USA), mRNA-1273 (NCT04283461, National Institute of Allergy and Infectious Diseases, USA), and SARS-CoV-2 rS (NCT04368988, Novavax). There are four vaccines on simultaneous Phase I-II: SARS-CoV-2 inactivated vaccine (NCT04352608, Sinovac Research and Development Co., Ltd.), LV-SMENP-DC (NCT04276896, Shenzhen Geno-Immune Medical Institute, China), ChAdOx1 (NCT04324606, University of Oxford, UK), and BNT162 (NCT04368728, Biontech SE, Pfizer). There is also one vaccine in Phase II: Ad5-nCoV (NCT04341389, Institute of Biotechnology, Academy of Military Medical Sciences, PLA of China) (47, 48). Of note, six peptide-based vaccines are currently under development: FlowVax™ by Flow Pharma Inc. (49), EPV-CoV19 by EpiVax (50), DPX-COVID-19 by IMV inc. (51), Vaxil Bio (US patent: 62/987,310) (52), OncoGen (53), and USask VIDO-InterVac (54).

In this urgent race to develop a vaccine, immunoinformatic techniques represent a powerful approach that allows the screening of whole pathogen proteomes to identify potential immunogenic regions (55). This includes predicting linear epitopes potentially presented by the Human Leukocyte Antigen (HLA) class I and II, which can be used to design peptide vaccines (56) and molecular diagnostic tests (57). The state-of-the-art programs are based on artificial neural networks (58–61), and they require both the sequence of target protein(s) and the host HLA allele(s) at 4-digit resolution (62, 63).

Two recent studies have extrapolated epitopes with experimental evidence in SARS-CoV to SARS-CoV-2, and selected candidates based on different criteria. Grifoni et al. (64) intersected these extrapolated epitopes with a set of predicted epitopes using 12 HLA-I A and B supertypes (65). They present 12 candidates in the S, M, and N proteins of SARS-CoV-2. Ahmed et al. (66) subselected the extrapolated epitopes using the Population Tool of the Immune Epitope Database and Analysis Resource (IEDB) (67), resulting in candidates in the S and N proteins that may potentially cover the global population. This tool is based on the HLA frequencies reported in the Allele Frequency Net Database (AFNDB), which is an important reference source for immunological studies worldwide (68). Nevertheless, this database has little information about South America, missing large studies published in recent years comprising millions of people (see Table S3).

Lack of knowledge about the HLA allelic distribution in South America can cause regional misrepresentation in immunological studies, which could result in diminished efficiency of vaccines and diagnostic tests. Additionally, knowledge on HLA allelic frequencies typified at 4-digit resolution or higher also plays a determinant role in other areas, like transplantation (69), response to cancer immunotherapy (70), and susceptibility to autoimmune diseases (71, 72).

Here, we performed a literature review of HLA allele frequencies of South American populations reported in scientific articles available in PubMed and datasets available in the AFNDB. These datasets were integrated by country, calculating weighted allele frequencies (WAFs). Thus, we are presenting updated WAFs for most South American countries. Then, HLA class I and II epitopes were predicted using only alleles with WAF ≥ 0.05. Finally, we selected candidate epitopes covering all of these South American HLA alleles, reporting both candidates with existing experimental evidence in the IEDB database for other coronaviruses as well as novel candidates. These candidates complement those proposed in recent articles, which in most cases scarcely cover South America. Our findings may result in a better representation of South America, enriching current development efforts of vaccines and diagnostic tests.

## Methods

### Alignment, Entropy, and Selection Pressure of the SARS-CoV-2 Proteins

A total of 2,123 genome sequences from human hosts, categorized as complete with high coverage in the Global Initiative on Sharing All Influenza Data (GISAID) database (73), were downloaded on March 31, 2020. This comprises genomes from 55 countries, including 16 from Brazil, seven from Chile, and one from Peru (Tables S1, S2). These sequences were aligned in CLC Main Workbench v.20.0.3 (QIAGEN Bioinformatics). The coding regions corresponding to the viral proteins were extracted and translated, using as reference the sequences with GenBank Gene IDs: 43740568 (Spike, S), 43740571 (Membrane, M), 43740575 (Nucleocapside, N), 43740570 (Envelope, E), 43740569 (ORF3a), 43740572 (ORF6), 43740573 (ORF7a), 43740574 (ORF7b), 43740577 (ORF8), 43740576 (ORF10), and 43740578 (Orf1ab and NSP1-16 proteins).

Variability at each amino acid position of the SARS-CoV-2 proteins was measured by Shannon Entropy (74), using the Shannon Entropy-One online tool (https://www.hiv.lanl.gov/content/sequence/ENTROPY/entropy_one.html). Sites with positive selection pressure were obtained from the SARS-CoV-2 Natural Selection Analysis available in the Galaxy Project (https://covid19.galaxyproject.org/) (75), retrieved on May 3, 2020, considering the better-ranked sites (meeting at least four categories).

### South American HLA Alleles and Weighted Allele Frequencies (WAFs)

A review was performed considering first only datasets available in the AFNDB containing allelic frequencies of HLA-A, -B, -C, -DPA, -DPB, -DQA, -DQB, and -DRB1 in South American populations. This was called the “current scenario.” We selected studies using the following inclusion criteria: (i) we collected all the studies characterizing HLA alleles with at least 4-digit resolution in 100 or more individuals (N ≥ 100); and (ii) an auxiliary rule was exceptionally applied in low-information cases only, i.e., if just two or fewer articles were obtained, both barely passing the filter (100 ≤ N ≤ 200). This exception consisted of relaxing the lower cut-off to N ≥ 40, allowing smaller studies to pass the filter to rescue additional data.

To expand the “current scenario,” in addition to the previously described datasets, we collected articles in PubMed reporting Class I and Class II HLA alleles of South American populations using the same inclusion criteria. This was called the “updated scenario.” The selection criteria were applied to both published datasets in scientific articles and datasets available only in the AFNDB. All alleles were matched to the current HLA nomenclature (76).

**Figure 1:**
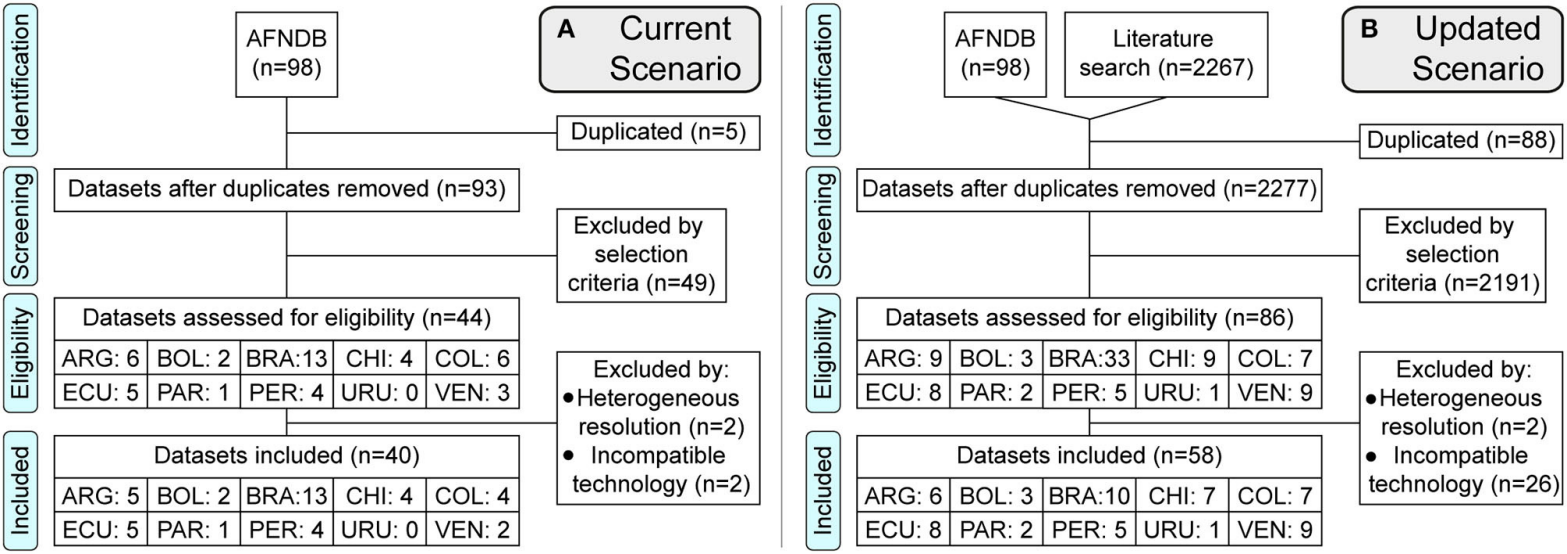
PRISMA flowchart of the literature review. Flow diagram of the datasets processed. **(A)** Current Scenario, using only the datasets available in the Allele Frequency Net Database. **(B)** Updated Scenario, adding databases from scientific literature until April 10, 2020. Countries are represented by the first three letters of their names. PRISMA checklist included in the Supplementary Material (77).

Then, for both current and updated scenarios, we calculated Weighted Allele Frequencies (WAFs) by country, using as approximation the weighted average of the allele frequencies in all the studies selected by country. This led to a few exceptions due to evident discrepancies in technology and resolution, resulting in the exclusion of some studies (see Figure 1 and Supplementary Methods for full detail). Only alleles with WAF ≥ 5% in the updated scenario were considered for further analysis.

### Epitope Prediction and Selection

Linear epitopes for HLA class I (HLA-A, -B, and -C) and HLA class II (HLA-DRB1) were predicted in the SARS-CoV-2 proteins using the alleles obtained in the previous step and the cut-offs recommended by each software. For HLA class I, we predicted epitopes of 8-11aa using NetMHCpan v4.0 (59) with rank ≤ 2 and MHCflurry v1.6.0 (60) with an affinity (IC50) ≤ 500 nM. As these programs do not have the same collection of HLA alleles available for prediction, we used the consensus verdict whenever possible. Otherwise, we only used the prediction of the software with the allele available. For HLA class II, we predicted epitopes of 15aa using NetMHCIIpan v4.0 (61) with rank ≤ 10. Then, we annotated which predicted epitopes have previous experimental evidence for other coronaviruses (identical match, either in full length from end to end or contained inside) in the IEDB Database (www.iedb.org) (67).

Each predicted epitope obtained corresponds to one or more HLA alleles, which have different WAFs by country. To combine these WAFs into a single value, we defined a Coverage Score (CS), which reflects how good a candidate epitope is for South America. This was calculated for each predicted epitope, by adding the proportion by country of alleles with WAF ≥ 5% that bind this predicted epitope. Therefore, this CS varies in a range from 0 to P, where P is the number of countries. Then, we selected candidate epitopes with the highest Coverage Scores, with and without prior experimental evidence. Additionally, for HLA-II only, we prioritized candidates based on their three-dimensional exposure. Sequence logos were generated using WebLogo 3 (78), showing the chemistry and frequency per amino acid of our best candidates.

All the data was processed and analyzed using Python v3.8.2 (www.python.org) and R v3.6.3 (www.r-project.org) with Rstudio v1.2.5033 (www.rstudio.com).

### Prediction of Post-translational Modifications Events

Signal peptide was predicted using Signal-3L 2.0 (79). Protein topology (inner, transmembrane, and outer regions) were predicted with MemBrain v3.1 (80). N-Glycosylation and O-Glycosylation sites were predicted using N-Glycosite (81) and NetOGlyc v4 (82) (score ≥ 0.5), respectively. Palmitoylation and Sumoylation sites were predicted using CSS-Palm 4.0 (83) (medium threshold, Sn = 86.92%, Sp = 89.97%) and GPS-SUMO v1.0 (84) (medium threshold, Sn = 68.94%, Sp = 95.01%), respectively. Prediction of ADP-ribosylation sites was performed using ADPredict v1.1 (85) (score ≥ 0.4). All predictions were manually curated based on Uniprot available annotations for SARS-CoV-2.

### Structural Modeling and Graphical Representation

Candidate epitopes were mapped on the 3D structure of the S protein. To avoid missing residues in the current crystal structure, we modeled the consensus sequence of the S protein by homology using the SWISS-MODEL web server (86) (https://swissmodel.expasy.org/), with the crystal structure as template (Protein Data Bank ID: 6VXX). Figures were generated in PyMOL v2.3.4 (https://pymol.org/2/) (87).

## Results

### Alignment, Entropy, and Selection Pressure of the SARS-CoV-2 Proteins

Diversity at each amino acid position revealed high entropy values in the proteins NSP2 (T85I, score = 0.36), NSP5 (L37F, score = 0.42), NSP12 (P323L, score = 0.69), NSP13 (P504L, score = 0.46 and Y541C, score = 0.47), Spike (D614G, score = 0.69), ORF3a (Q57H, score = 0.41 and G251V, score = 0.31), ORF8 (L84S, score = 0.57), and Nucleocapsid (R203K, score = 0.41, and G204R, score = 0.41).

Sites with the highest probability to be under positive selective pressure are located in NSP2 (T85, P568), NSP3 (K384, N444, P822, V1768, V1795), NSP6 (L75), NSP7 (S25), Spike (S943, G1124), ORF3a (A99, T14, L147), NSP12 (A97, L323, A449), NSP13 (V49), NSP14 (A482), and NSP16 (K160). See **Figure 3** and Table S8.

### South American HLA Alleles and Weighted Allele Frequencies (WAFs)

We found 44 eligible datasets for the “current scenario” and 86 for the “updated scenario”, using only 40 and 58 to calculate the WAFs by country, respectively (Figure 1). The selection process is provided in full detail in the Supplementary Methods and Table S3.

The IEDB population coverage tool (http://tools.iedb.org/population/) uses information provided by the AFNDB at 4-digit resolution, which in the case of South America comprises just a small number of populations: two from Argentina, one from Bolivia, five from Brazil, two from Chile, three from Colombia, two from Ecuador, one from Paraguay, two from Peru, and four from Venezuela. For the current scenario, we collected a similar number of datasets: five from Argentina, two from Bolivia, 13 from Brazil, four from Chile, four from Colombia, five from Ecuador, one from Paraguay, four from Peru, and two from Venezuela. In both cases, there was no data for Uruguay, Guyana, French Guiana, or Suriname.

Our literature review to update the allele frequencies included six datasets for Argentina, three for Bolivia, 10 for Brazil, seven for Chile, seven for Colombia, eight for Ecuador, two for Paraguay, five for Peru, one for Uruguay, and nine for Venezuela. In both scenarios (current and updated), we only obtained HLA-II and not HLA-I data from Bolivia. All of the allele frequencies and sample sizes by study are provided in Table S4. The addition of new studies resulted in updated HLA allele frequencies.

**Figure 2:**
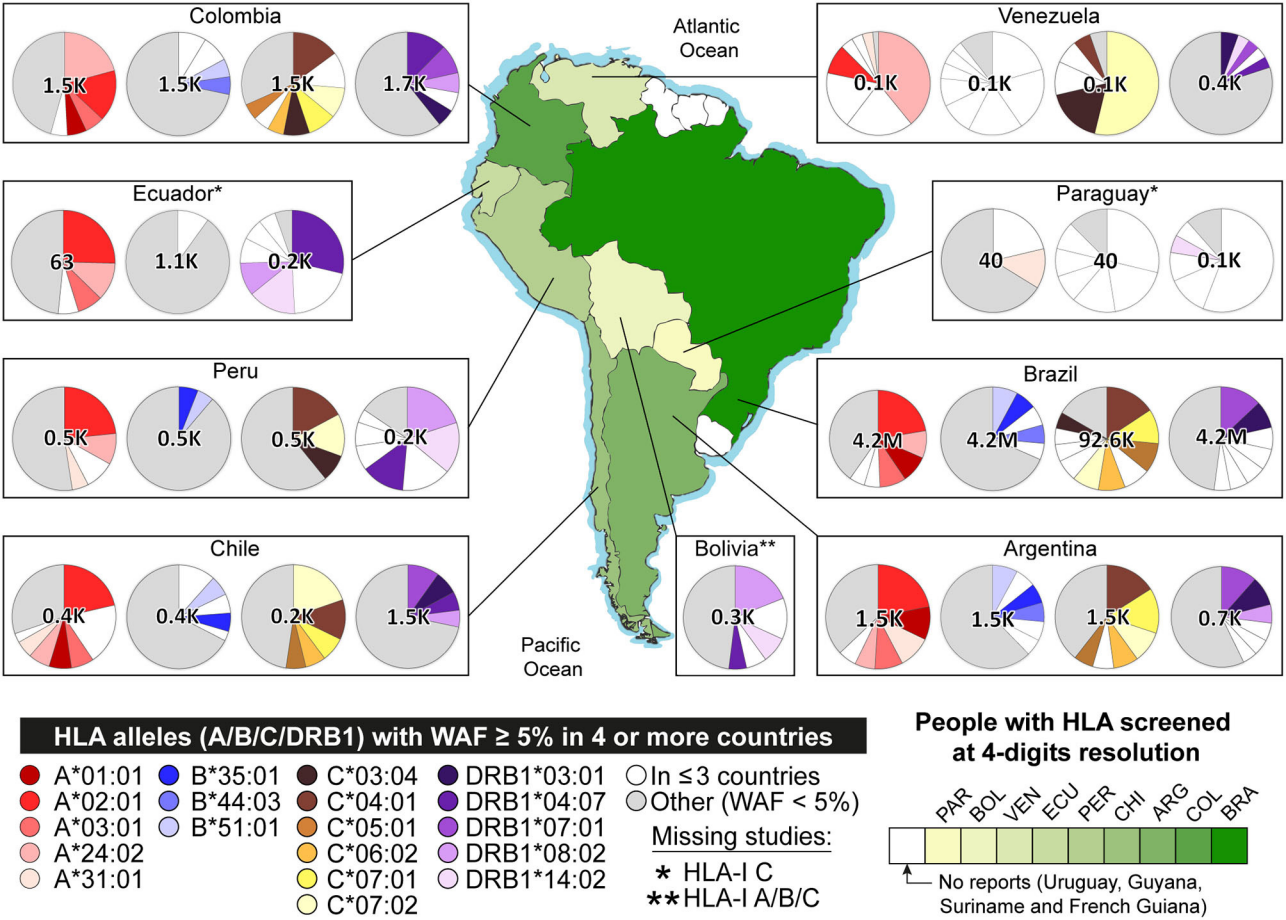
Allele frequencies of HLA-I A, B, C, and HLA-II DRB1 in South America. The pie charts represent the distribution of allele frequencies in genes HLA-I A, B, C, and HLA-II DRB1. Alleles with Weighted Allele Frequency (WAF) ≥ 5% in four or more countries are represented in scales of red (A), blue (B), brown (C), and purple (DRB1), respectively. Alleles with WAF ≥ 5% in three or fewer countries are shown in white. Alleles with WAF < 5%, are shown in gray. The green gradient represents the extent of people with HLA alleles genotyped. Detailed data is available in Tables S5, S6.

We then calculated WAFs for each country (see Table S5). Some alleles with WAF under 5% in the current scenario are now above in the updated scenario: 13 alleles of Argentina, 15 of Brazil, seven of Chile, seven of Ecuador, one of Paraguay, two of Peru, and 19 of Venezuela. Additionally, some alleles not reported in the AFNDB for South America were found with WAF ≥ 5% in the updated scenario: six alleles of HLA-I C in Argentina, two HLA-II DQA1 in Ecuador, two HLA-I A and six HLA-I B in Paraguay, two HLA-I C in Peru, five HLA-II DQB1 in Uruguay, and four HLA-II DPA1 in Venezuela (see Figure 2). Details provided in Table S6.

### Epitope Prediction

We obtained 11,644 predicted T cell epitopes in SARS-CoV-2 proteins: 7,517 for HLA-I and 4,127 for HLA-II. We found that 1400 have previous experimental evidence in the IEDB: 25 in positive T cell assays, 1327 in positive MHC-ligand binding assays, and 48 in both (see Table S7).

In the S protein, we predicted 961 HLA-I epitopes. Previous experimental evidence was found for 113, although they had low CS (0.048–2.146). The best two predicted epitopes with the highest CS (VVFLHVTYV, CS = 2.146, IEDB-ID: 71663 and LQIPFAMQM, CS = 2.043, IEDB-ID: 38855) cover almost one allele with WAF ≥ 5% by country. These were previously proposed as candidates by Ahmed et al., 2020 (66). Other predicted epitopes already proposed as candidates in similar studies (64, 66) have lower CS. From the 848 predicted epitopes without experimental evidence, the top three with the highest CS cover at least one allele with WAF ≥ 5% by country. These represent novel candidates (Table 1): MIAQYTSAL (CS = 4.127), SIIAYTMSL (CS = 3.739), and YLQPRTFLL (CS = 3.646).

**Table 1:** Best HLA-I candidate epitopes for South America in SARS-CoV-2 proteins.

**Best HLA-I candidate epitopes for South America**						**N° of alleles with WAF ≥ 0.05 covered by country**									**Coverage score**	**Reference**
**Protein**	**Start**	**End**	**Peptide**	**Experiment**	**IEDB ID**	**ARG**	**BOL**	**BRA**	**CHI**	**COL**	**ECU**	**PAR**	**PER**	**VEN**		
S	1060	1068	VVFLHVTYV	LTM, ML	71663	3	-	5	3	6	1	2	3	9	2.146	Ahmed et al. (66)
	894	902	LQIPFAMQM	ML	38855	1	-	4	2	5	0	6	1	11	2.043	
ORF6	3	11	HLVDFQVTI	ML	24313	3	-	6	4	6	1	3	3	12	2.520	New
NSP3	950	958	VMYMGTLSY		5477, 70040	6	-	7	5	7	1	5	2	8	2.801	
NSP5	219	227	F**L**NRFTTTL		16786	6	-	9	6	8	1	3	4	13	3.224	
NSP6	86	94	MPASWVMRI		42260, 42261	4	-	6	5	6	0	3	3	9	2.292	
NSP8	47	55	SEFDRDAAM		57419	3	-	2	3	4	1	5	0	7	1.832	
NSP12	877	885	YADVFHLYL	ML	14969	6	-	11	7	8	1	3	5	10	3.346	New
	123	131	TMADLVYAL		65176, 65177	3	-	5	4	6	1	4	4	13	2.756	
	898	906	HMLDMYSVM		24342	3	-	5	3	5	1	4	4	12	2.591	
NSP13	355	363	YVFCTVNAL	ML	76266	6	-	9	6	8	1	3	5	13	3.335	New
	291	299	FAIGLA**L**YY		23758	6	-	9	7	8	1	3	3	6	2.838	
NSP14	494	502	YLDAYNMMI	ML	74593	5	-	9	6	8	1	1	4	11	2.823	New
	500	508	MMISAGFSL		42128	2	-	5	4	6	1	4	3	13	2.590	
S	869	877	MIAQYTSAL			8	-	10	8	9	1	5	6	15	4.127	New
	691	699	SIIAYTMSL	-	-	6	-	8	6	9	1	5	5	16	3.739	
	269	277	YLQPRTFLL			6	-	8	6	8	2	3	6	14	3.646	
NSP2	420	428	YITGGVVQL	-	-	5	-	8	6	9	1	5	4	12	3.382	New
	265	273	GLNDNLLEI			2	-	4	2	5	1	1	3	7	1.705	
NSP3	1776	1784	YVNTFSSTF			6	-	8	7	9	1	4	4	10	3.276	New
	1452	1460	YLNSTNVTI	-	-	5	-	7	6	9	1	3	4	14	3.180	
	816	824	YYHTTD**P**SF			6	-	9	8	9	1	2	5	8	3.148	
NSP4	25	33	YLITPVHVM	-	-	6	-	7	6	9	1	6	5	14	3.721	New
	309	317	**G**EYSHVVAF			3	-	3	3	5	1	6	1	9	2.270	
NSP12	442	450	FAQDGNA**A**I	-	-	8	-	12	8	10	1	4	6	12	4.013	New
	281	289	KLFDRYFKY			4	-	4	4	4	1	5	2	5	2.169	
NSP16	103	111	FVSDADSTL	-	-	6	-	11	7	9	1	3	4	13	3.437	New

*These candidate epitopes were selected from our prediction based on their scores (rank for NetMHC and IC50 for MHCflurry) and Coverage Score (CS). Experimental evidence including the IEDB ID and experiment type (LTM: linearT_MHC, ML: MHC_ligand) is provided when available. Scientific articles already proposing these candidates are mentioned. Residues in bold represent positive selection pressure sites*.

We predicted 6556 HLA-I epitopes in other SARS-CoV-2 proteins. Experimental evidence was reported for 1122, and the 10 with the highest CS (2.520–3.346) cover at least one allele with WAF ≥ 5% by country. These predicted epitopes are located in the ORF6 and NSP proteins, and represent novel candidates. The highest CS (3.346) corresponds to YADVFHLYL, located in the NSP12 protein. We also found 5434 predicted epitopes without experimental evidence. The top seven with the highest CS (3.148–4.013) are novel candidates, located in NSP proteins, and cover at least one allele with WAF ≥ 5% by country. The candidate with the highest CS (FAQDGNAAI, 4.013) is also located in the NSP12 protein (see Table 1).

**Figure 3:**
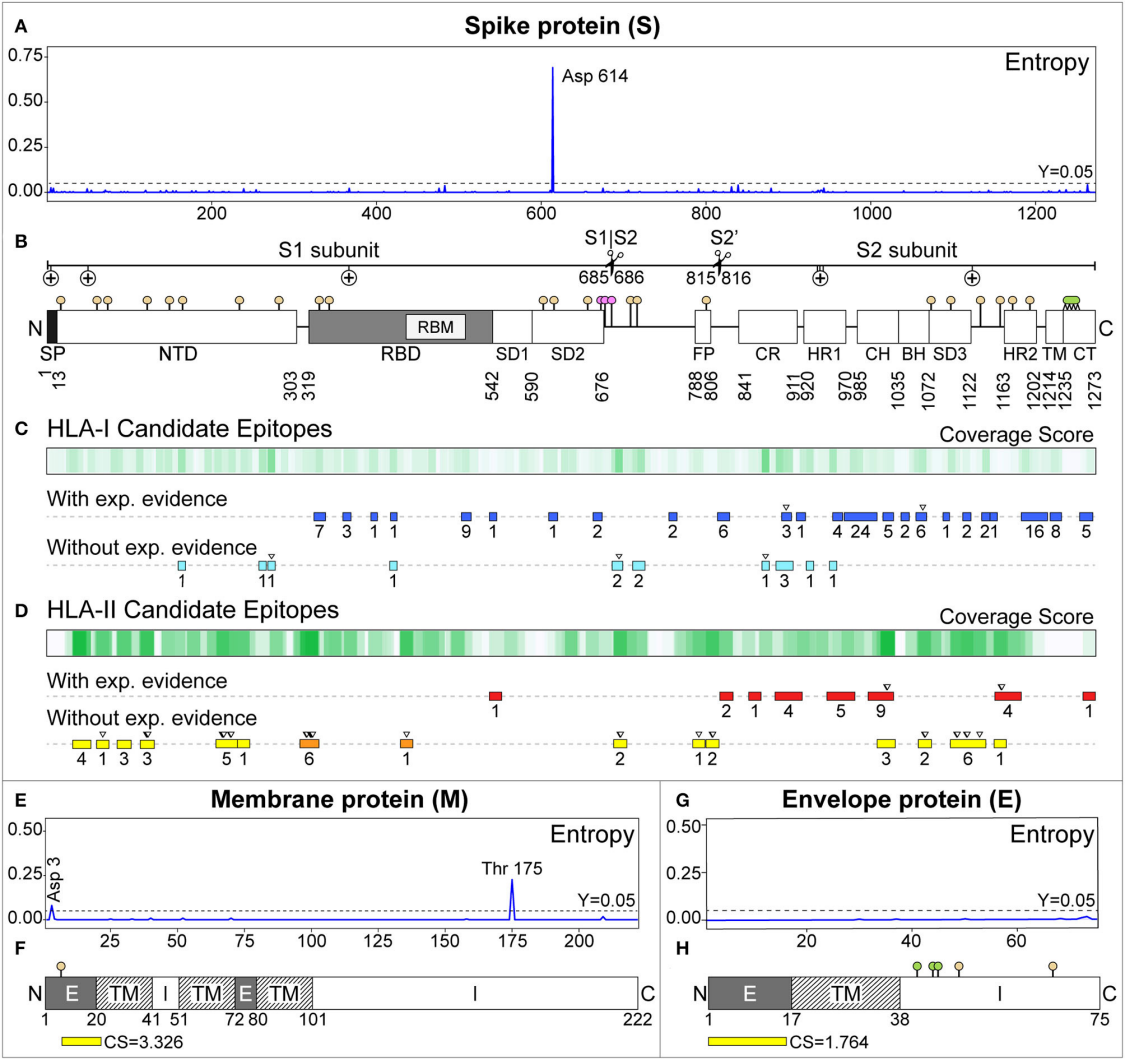
Candidate epitopes in the sequence of the Membrane (M) and Spike (S) proteins of SARS-CoV-2. **(A,E,G)** Show the entropy per amino acid for the S, M, and E proteins, respectively, calculated by aligning 2123 SARS-CoV-2 genomes. In **(B,F,H)**, post-translational modifications are represented as sticks with colored circles: beige (N-linked glycosylations), pink (O-GalNAc glycosylations), and lemon (palmitoylations). **(B)** Regions of the S protein, indicating the subunits 1 (S1), 2 (S2), and cleavage points (scissors). Positive selection pressure is represented with (+). SP, Signal peptide; NTD, N-terminal domain; RBD, Receptor Binding Domain; RBM, Receptor Binding Motif; SD1, Sub-Domain 1; SD2, Sub-Domain 2; FP, Fusion Peptide; CR, Connecting Region; HR1, Heptad Repeat 1; CH, Central Helix; BH, B-Hairpin; SD3, Sub-Domain 3; HR2, Heptad Repeat 2; TM, Transmembrane domain; CT, Cytoplasmic tail. **(C)** HLA-I epitopes predicted for South American alleles with WAF ≥5%. The gradient of green represents the coverage scores. The rectangles below represent the predicted epitopes with experimental evidence in the IEDB (blue), and those without experimental evidence with CS ≥2 (light blue). Overlapping predicted epitopes are represented by a single rectangle with the number of epitopes contained (underneath). The inverted triangle on top highlights our best candidates. **(D)** Analogously, for HLA-II. Predicted epitopes with experimental evidence are shown in red. Without experimental evidence and CS ≥6, in yellow. Those in the RBD are highlighted in orange. **(F,H)** Represent the topology of the M and E proteins, respectively. Exposed (E), transmembrane (TM), and intra-virion (I) regions were extracted from annotated proteins (UniProtKB IDs: P0DTC4 and P0DTC5). The best HLA-II epitopes predicted in their exposed regions (and their CS) are shown in yellow.

For HLA-II, we predicted 628 epitopes in the S protein. Twenty-eight have experimental evidence, and the two with the highest CS (RAAEIRASANLAATK, CS = 9.000, IEDB-ID: 100428 and IRAAEIRASANLAAT, CS = 8.148, IEDB-ID: 100428) are contiguous and belong to the CH region. They represent novel candidates (see Table 2). The next two, LDKYFKNHTSPDVDL (CS = 6.760, IEDB-ID: 35205) and DKYFKNHTSPDVDLG (CS = 6.760, IEDB-ID: 9006), are also contiguous and correspond to the HR2 region (Figure 3D). These two were selected as candidates in a previous study (66).

**Table 2:** Best HLA-II candidate epitopes for South America in SARS-CoV-2 proteins.

**Best HLA-II candidate epitopes for South America**							**N° of alleles with WAF ≥ 0.05 covered by country**									**Coverage score**	**References**
**Protein**	**Start**	**End**	**Peptide**	**Region**	**Experimental**	**IEDB ID**	**ARG**	**BOL**	**BRA**	**CHI**	**COL**	**ECU**	**PAR**	**PER**	**VEN**		
S	1013	1027	IRAAEIRASANLAAT	CH	LTM	100428	5	5	5	4	5	7	4	7	4	8.148	New
	1014	1028	RAAEIRASANLAATK			100428	6	5	7	4	5	7	5	7	5	9.000	
	1152	1166	LDKYFKNHTSPDVDL	HR2	ML	35205	4	4	4	3	4	5	5	6	3	6.760	Ahmed et al. (66)
	1153	1167	DKYFKNHTSPDVDLG			9006	4	4	4	3	4	5	5	6	3	6.760	
S	61	75	NVTWFHAIHVSGTNG	NTD	-	-	4	4	4	3	4	5	4	4	4	6.474	New
	114	128	TQSLLIVNNEATNVVI				4	4	5	3	4	6	5	6	3	7.045	
	115	129	QSLLIVNNATNVVIK				5	4	6	4	5	5	5	5	4	7.719	
	116	130	SLLIVNNATNVVIKV				4	3	5	3	4	4	5	4	4	6.474	
	206	220	KHTPINLVRDLPQGF				4	3	4	3	4	5	3	5	3	6.017	
	207	221	HTPINLVRDLPQGFS				5	4	5	3	4	6	5	6	4	7.412	
	208	222	TPINLVRDLPQGFSA				4	4	3	3	4	5	2	6	3	6.017	
	216	230	LPQGFSALEPLVDLP				3	4	3	3	3	5	5	6	4	6.450	
	217	231	PQGFSALEPLVDLPI				3	4	3	3	3	5	5	6	4	6.450	
	308	322	VEKGIYQTSNFRVQP	RBD	-	-	4	5	5	3	4	7	5	7	3	7.531	
	309	323	EKGIYQTSNFRVQPT				5	5	6	4	5	7	5	7	4	8.490	
	313	327	YQTSNFRVQPTESIV				3	5	3	3	3	6	5	6	3	6.593	
	314	328	QTSNFRVQPTESIVR				6	5	7	4	5	6	5	6	5	8.714	
	315	329	TSNFRVQPTESIVRF				6	5	7	4	5	7	5	7	5	9.000	
	316	330	SNFRVQPTESIVRFP				3	4	5	3	3	5	5	5	4	6.593	
	430	444	TGCVIAWNSNNLDSK				4	4	6	3	4	6	5	6	4	7.388	
	689	703	SQSIIAYTMSLGAEN	-	-	-	3	5	3	3	4	7	4	7	3	6.879	
	690	704	QSIIAYTMSLGAENS				4	5	4	3	4	7	5	7	3	7.388	
	785	799	VKQIYKTPPIKDFGG				3	5	2	3	3	5	4	6	3	6.107	
	801	815	NFSQILPDPSKPSKR	FP	-	-	5	4	5	3	4	6	4	6	4	7.212	
	802	816	FSQILPDPSKPSKRS				5	4	5	3	4	6	5	6	4	7.412	
	1059	1073	GVVFLHVTYVPAQEK	BH	-	-	3	5	3	3	3	7	5	7	4	7.079	
	1060	1074	VVFLHVTYVPAQEKN				3	4	2	3	3	5	4	6	4	6.107	
	1098	1112	NGTHWFVTQRNFYEP	SD3	-	-	4	4	4	3	3	5	5	5	4	6.617	
	1099	1113	GTHWFVTQRNFYEPQ				4	4	4	3	3	6	5	6	4	6.902	
	1110	1124	YEPQIITTDNTFVS**G**				4	4	4	3	4	5	5	5	4	6.817	
	1111	1125	EPQIITTDNTFVS**G**N				4	4	6	3	4	6	5	6	4	7.388	
	1126	1140	CDVVIGIVNNTVYDP	-	-	-	4	3	5	3	4	4	5	4	4	6.474	
M	7	21	TITVEELKKLLEQWN	Virion Surface	-	-	2	2	2	1	1	3	3	3	2	3.326	New
E	1	15	MYSFVSEETGTLIVN	Virion Surface	-	-	0	2	1	1	1	2	0	2	1	1.764	New

*These candidate epitopes were selected from our prediction based on their scores (rank for NetMHC and IC50 for MHCflurry), Coverage Score (CS), and exposure (for Class-II only). Experimental evidence including the IEDB ID and experiment type (LTM: linearT_MHC, ML: MHC_ligand) is provided when available. Scientific articles already proposing these candidates are mentioned. Underlined residues indicate sites with predicted N-linked glycosylations. Residues in bold represent positive selection pressure sites*.

**Figure 4:**
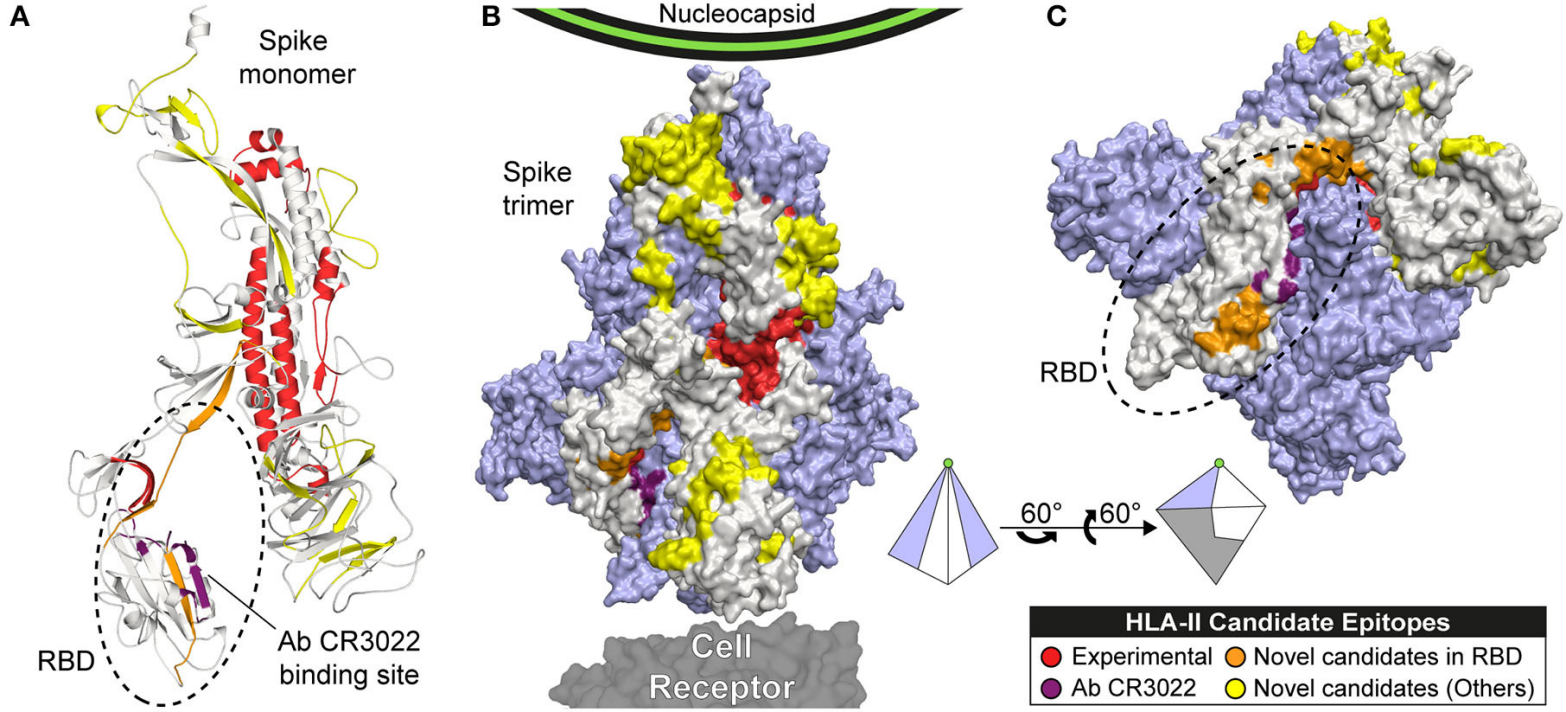
Candidate epitopes in the 3D structure of the Spike (S) protein of SARS-CoV-2. This figure shows our HLA-II candidate epitopes with experimental evidence (red), without experimental evidence and CS ≥ 6 (orange: located in RBD, yellow: located in other regions), and the binding site of the Ab CR3022 (violet). Candidate epitopes were mapped to the Spike monomer **(A)**, and the Spike trimer (**B** and **C**). The monomer is represented in beige, and the other 2 subunits in light purple. **(C)** Rotation of the trimer showing the RBD region. A pyramid representing the trimer is shown as visual aid to represent the rotation.

We also predicted 601 epitopes without experimental evidence in the S protein, where 41 had 6.017 ≤ CS ≤ 9.000 (Figure 3D). Some of these are located in notoriously exposed regions (see Figure 4). All of these are novel candidates, including EKGIYQTSNFRVQPT (CS = 8.490), QTSNFRVQPTESIVR (CS = 8.714), and TSNFRVQPTESIVRF (CS = 9.000, the maximum possible score), which overlap in the RBD and have a remarkably high CS.

We obtained 81 HLA-II predicted epitopes in the M protein. Only one (PKEITVATSRTLSYY, CS = 6.110) has a reported experiment (IEDB-ID: 48051), an MHC-ligand assay presenting the peptide to the HLA-DRB1^*^01:01, measuring the affinity. It was already selected as a candidate in another study (66). Nevertheless, it is located in a predicted intra-virion region. Of those without experimental evidence and located outside the virion, TITVEELKKLLEQWN has the best CS (3.326) (see Figure 3F). In the E protein, we predicted 15 HLA-II epitopes. Similarly, only LVTLAILTALRLCAY has previous experimental evidence, but a very low CS (0.143), and it is located in a predicted non-external region. However, the candidate MYSFVSEETGTLIVN (CS = 1.764) is located outside the virion (Figure 3H). Sequence logos of our class-I and class-II candidate epitopes are presented in Figures S1, S2, respectively.

To cover all the HLA alleles with WAF ≥ 5% in South America, we included five additional candidates. We selected predicted epitopes that bind to HLA alleles not covered by any of the candidates already selected, choosing those with the highest CS. Two have experimental evidence: MPASWVMRI (in NSP6, CS: 2.292, IEDB ID: 42260 and 42261) and SEFDRDAAM (in NSP12, CS:1.832, IEDB ID: 57419). The remaining three have no experimental evidence: GEYSHVVAF (in NSP4, CS: 2.270), KLFDRYFKY (in NSP12, CS: 2.169) and GLNDNLLEI (in NSP2, CS: 1.705).

### Prediction of Post-translational Modifications Events

Twenty two potential N-linked glycosylation sites were predicted along the S protein in three clusters: (i) inside the NTD and RBD regions (N17, N61, N74, N122, N149, N165, N234, N282, N331, and N343); (ii) in the proximity of the S1/S2 and S2' cleavage sites (N603, N616, N657, N709, N717, and N801); and (iii) near the C-terminus (N1074, N1098, N1134, N1158, N1173, and N1194). In the M protein, one predicted N-linked glycosylation site (N5) is located in the exposed region, and it has been associated with antigenicity and transport in some coronaviruses (88). The E protein presents two potential N-linked glycosylation sites: N48, probably non-functional (89), and N66, suggested in SARS-CoV to be potentially associated with monomeric forms (90). ORF8 also presents a potential N-linked glycosylation site in N78, which could stabilize and protect the protein from proteasomal degradation, as occurring in SARS-CoV (91). O-linked glycosylations were predicted in residues S673, T678, and S686 of the S protein and residues T32 and T34 of the ORF3a. These events were experimentally detected in SARS-CoV (92). However, glycosylation in residues 686 (O-linked) and 1158 (N-linked) were not confirmed by mass spectrometry in a recent study (93).

Then, the predicted glycosylation sites were contrasted with the best HLA-II candidate epitopes for South America (Table 2). Sites N61, N122, N801, N1074, N1098, and N1158 are located in nine HLA-II candidate epitopes. Additionally, sites N234, N331, N709, and the O-linked 686 fall near eight candidate epitopes.

Predicted palmitoylation sites fall into the cytoplasmic tail (C1235, C1236, C1240, C1243, C1247, C1248, C1250, C1253, and C1254) of the S protein and three cysteines (C40, C43, and C44) of the E protein. These sites have been previously reported in SARS-CoV (94), being associated with protein subcellular trafficking, stability and viral assembly (95, 96).

In the N protein, a potential sumoylation site was predicted in K338. Li et al. (97) explored sumoylation events experimentally in SARS-CoV, not finding K338 but detecting the site K62. This site was also detected in our prediction using a less restrictive threshold. This corresponds to K61 in SARS-CoV-2, having an Asp instead of Glu in the canonical consensus motif. This site has been associated with self homo-oligomerization and host cell division interference.

ADP-ribosylation prediction identified eight potential sites along the nucleocapsid (D22, E118, E136, E231, E253, E323, E378, and D415). This PTM was also reported in other coronaviruses, and can be related to the virus infective phase (33) (see Figure 3 and Table S8).

### Structural Modeling of the S Protein

The monomer model generated is composed of the first 1147 residues of the SARS-CoV-2 Spike protein. It fills the gaps of the crystal structure PDB:6VXX at positions 1-26, 70-79, 144-164, 173-185, 246-262, 445-446, 455-461, 469-488, 502, 621-640, 677-688, and 828-853.

HLA-II candidate epitopes shown in Figure 3 were mapped to the trimeric 3D-structure in order to visualize their exposure (Figure 4). However, residues 1148-1273 were not represented in our model as they are missing in the reference crystal (22). Candidates in this missing region are represented in Figure 3D only.

## Discussion

There is an urgent need to develop vaccines and better diagnostic tests for COVID-19, targeting specific immunogenic regions and epitopes with protective potential and population representativeness. Our research presents an updated report of HLA genotypes of South American populations, which led to a selection of candidate epitopes for HLA class I and II supported by experimental evidence as well as novel candidates, predicted to cover all South American countries.

The AFNDB and the IEDB population coverage tools are frequently used by the scientific community worldwide as reference sources of HLA frequencies (68), meaning it is crucial to have them updated. However, their collection and curation of new data rely on the scientific community users (68, 98). For South America, these databases contain mostly small datasets coming from ethnic groups, not representative of the countries' diversity, resulting in a current inaccurate distribution of HLA frequencies (see Table S6). We have found 30 large datasets from 10 South American countries which were not included in the AFNDB. Our literature review represents a large update from a scenario of 20,124 to now 12,857,200 datapoints among all the alleles collected of HLA-A, -B, -C, -DPA1, -DPB1, -DQA1, -DQB1, and -DRB1. This is reflected in 86 HLA alleles with WAF ≥ 5% in the updated scenario, which were previously considered with frequencies under 5% or missing in the current scenario. This issue results in a misrepresentation of South America that could be affecting multiple immunological studies using these sources, like Ahmed et al. (66) and other SARS-CoV-2 recent studies in the pre-print stage. To encourage and facilitate using the information collected, we are providing the datasets by country in their full extent (Tables S3, S4) and averaged (Tables S5, S6), as well as the selection and matching process in full detail (Supplementary Methods).

We are also presenting weighted allele frequencies by country, providing a one-sight representation of South America HLA abundances (Figure 2). This clearly shows that some HLA genes are understudied, especially in countries like Venezuela, Bolivia, and Paraguay. Moreover, there is no data from Uruguay, Guyana, French Guiana, or Suriname (68). We recommend genotyping the HLA of large populations to reduce diversity misrepresentation, like the study of the Brazilian bone marrow registry (99), which provided HLA alleles of millions of people.

Epitopes were predicted using MHCflurry v1.6.0 (60), NetMHCpan v4.0 (59), and NetMHCIIpan v4.0 (61). These state-of-the-art software are based on neural networks and use binding, stability, and eluted MHC-ligand mass spectrometry data (58). The recent update of NetMHCIIpan from v3.2 to v4.0 represents an evident improvement in prediction accuracy, being necessary to use the last version. We enriched our predictions, adding the experimental evidence reported in the IEDB for other coronaviruses. Nevertheless, certain class-II potential epitopes found in binding experiments (i.e., measuring the affinity of epitopes presented *in-vitro* to the MHC molecule) were located in transmembrane or internal regions. As the conformational dynamics of SARS-CoV-2 proteins remains unknown, we opted for being extra cautious, selecting only class-II candidate epitopes in known exposed regions of the protein structure.

We then define a Coverage Score (CS), a metric representing to what extent a candidate epitope covers the HLA alleles of South America. As this score is based on alleles and not on haplotypes, it might be overestimating the actual coverage. However, it represents a reasonable approximation for the current available data, mostly reported as allelic frequencies. Based on the CS, we are presenting the best predicted epitopes, with and without experimental evidence, as potential candidates. Remarkably, we found novel candidates with very high CS, some located in immunologically relevant regions like the RBD of the Spike protein. And exposed regions of the M and E proteins (Figures 3, 4).

Viral clearance of SARS-CoV-2 infection requires activating subsets of CD4^+^ and CD8^+^ T cells (100). Whereas, HLA-I epitopes are derived from both structural and non-structural proteins due to their endogenous processing, HLA-II epitopes have exogenous processing, being structural proteins (like S, M, or E) of particular interest (64). In SARS-CoV, CD8^+^ T cell response was previously shown to be greater than CD4^+^, and it is widely elicited by different proteins including the replicase (ORF1ab) and NSPs (101). Even though structural proteins are associated with stronger T-cell responses (102–104), non-structural proteins can also induce an immune response and provide additional epitopes (101). Similar experimental studies are needed to corroborate the same behavior in SARS-CoV-2. In fact, we found several HLA-I candidate epitopes in NSP proteins with a high coverage of South American HLA alleles, with and without experimental evidence (Table 1).

Several studies had demonstrated the immunogenicity of protein S in beta-coronaviruses, being the main target for vaccine development (22, 105–109). Its receptor binding domain (RBD) interacts with the human receptor ACE2, playing a crucial role during the viral entry process (110). Therefore, antibodies binding this region could potentially impede viral recognition. We are presenting 32 HLA-II candidate epitopes in the S protein, including seven novel candidates located in the RBD and exposed in the 3D structure. This includes the candidate epitope TSNFRVQPTESIVRF, which covers all the HLA-II alleles of South America. Some of our candidates are located near the binding site of the monoclonal antibody CR3022 in the RBD (111, 112) (Figure 4C). This antibody neutralizes SARS-CoV (113), opening the possibility of finding neutralizing antibodies against our HLA-II candidate epitopes. Additionally, neutralizing monoclonal antibodies has been generated against S1 and S2 for SARS (104, 114), and against the RBD for SARS (115, 116) and MERS (117, 118).

Other domains of the S protein with predicted HLA-II epitopes are in accordance with previous evidence in other coronaviruses. It has been shown that the fusion peptide, the HR1 region, and the central helix are potential targets for broadly neutralizing antibodies (119). We also found candidate epitopes located in exposed regions of the NTD, the Subdomain 3 (SD3), the B-hairpin (BH), the Central Helix (CH), the Heptad Repeat (HR2), and linker regions, which could serve as potential antibody targets. Peptides derived from the Heptad regions HR1 and HR2 have proven to be effective inhibitors of viral fusion in SARS (120, 121) and MERS (122, 123). Moreover, conformational changes of the S protein trimer could explain the presence of cryptic class-II epitopes (not accessible in the canonical 3D structure) (112). This opens up the possibility of finding candidate epitopes in unexposed regions of the protein, as hinted by our predicted epitopes with experimental MHC-peptide binding evidence (Figure 4B).

Our HLA-I and HLA-II candidate epitopes cover 100% of the alleles with WAF ≥ 5% in South America. Remarkably, we obtained two HLA-II candidate epitopes with the maximum CS possible (covering all the alleles), one with experimental evidence.

Some of our predicted epitopes have been previously reported by other studies using similar approaches (Tables 1, 2). Our selection agrees with two candidate epitopes from Ahmed et al. (66). They collected experimentally-determined epitopes and the corresponding alleles against which these were tested. Then, they used the IEDB population coverage tool to select 87 candidate epitopes for one HLA-II and 32 HLA-I alleles in total, aiming to cover 96.29% of people worldwide. These alleles represent only 18/47 (38.3%) of the HLA-I and 1/18 (5.6%) of the HLA-II alleles with WAF ≥ 5% in our literature review for South America. Moreover, using the same tool with their candidates and alleles, but selecting South American populations only (according to the tool), we obtained a coverage of just 90.6% HLA-I and 4.1% HLA-II alleles. These comparisons were done using only the experimentally-determined binding alleles. Furthermore, we explored if their candidate epitopes could bind our alleles using our prediction methodology (i.e., calculating the affinity of their candidate epitopes to the South American HLA alleles with WAF ≥ 5%). However, we obtained a match of just 43/47 (91.49%, HLA-I) and 11/18 (61.11%, HLA-II). Altogether, this suggests a misrepresentation that leads to diminished coverage for South America.

Grifoni et al. (64) selected candidate epitopes in SARS-CoV-2 through: (i) sequence homology with epitopes with experimental evidence in SARS-CoV, and by (ii) epitope prediction, using 12 supertype representatives (the six most frequent HLA-I A and B alleles worldwide). Intersecting these two sources, they selected 12 HLA-I candidate epitopes in common, having an identity ≥ 90% with SARS-CoV. Their selection does not agree with any of our candidates. However, during their prediction, they obtained 12 HLA-I (CS: 0.095–3.739) and two HLA-II (CS: 6.474–6.593) predicted epitopes that have better scores for South America and match with some of our candidates. Nevertheless, further steps in their selection criteria made them drop these candidates. This is attributable to their strict filtering, due to the fact that they relied on experimental evidence from SARS-CoV only, as well as the small number of supertype alleles and epitopes chosen.

An important improvement in selecting the best candidate epitopes is to consider problematic sites affected by entropy, selective pressure, post-translational modifications, and other effects. Some of these considerations have been demonstrated to assist in developing molecular diagnosis in coronaviruses and other species (124, 125). Amino acid variants can result in diverse changes affecting the infectious and adaptive virus behavior. Entropy analysis revealed highly variable sites such as P323L in NSP12 (RdRp) or D614G in the S protein. Further biological consequences could be obtained from the sites affected by positive selective pressures, like T85 in NSP2, S25 in NSP7, and A99 in ORF3a. In the present study, we found that five of 27 HLA-I and two of 34 HLA-II candidate epitopes contain predicted sites affected by positive selective pressure (shown in bold in Tables 1, 2). However, we decided to keep these epitopes in our list of potential candidates due to their high CS and low amino acid variability (under 5%) in the current pandemic wave.

We predicted PTMs, including glycosylation, palmitoylation, sumoylation, and ADP-ribosylation events, in order to find relevant sites for the viral cycle. Sites affected by these events are expected to be conserved as they fulfill critical functions in viruses (33, 95–97). N-linked glycosylation is one of the most frequents PTMs with potential effects over the folding, tropism, interactions with host proteases, antibody recognition, and antigenicity of the Spike protein (126–131). N-linked glycosylations have been predicted in our candidate epitopes, including those with experimental evidence (Table S8). SARS-CoV-2 Spike protein possesses 22 potential N-linked glycosylation sites (Figure 3), mainly distributed in S1 and the C-terminal region of the S2 (119, 132). Some of them are located in the NTD and near the S1/S2 cleavage region (N122, N165, N234, N603, and N717). These sites surround the ACE2-binding domain and were shown to be critical for viral entry mediated by DC-SIGN (dendritic cell-specific ICAM-3-grabbing non-integrin) and L-SIGN (liver/lymph node-specific ICAM-3-grabbing non-integrin) (133), which are two C-type lectins that recognize high-mannose glycans (134). In contrast to SARS-CoV, SARS-CoV-2 presents an additional site (N657, near the S2 cleavage) and misses the glycosylation site N370 (in the RBD region), due to the absence of Ser/Thr to complete the sequon. Although this does not alter the affinity to the ACE2 receptor (135), it can significantly reduce DC-SIGN binding capacity (136). Thus, availability of N-linked glycosylations sites and differential affinities to ACE2, DC-SIGN, or L-SIGN may act as either enhancer forces or alternative mechanisms for viral entry (23, 137). Additionally, sites N227 and N699 in SARS-CoV (equivalent to N234 and N717 in SARS-CoV-2) have been hypothesized to facilitate the zoonotic transmission of this virus (133). A recent study based on liquid-chromatography-mass spectrometry (LC-MS) (132) confirmed the occurrence of N-linked glycosylation in all of our predicted sites. However, another study using LC-MS/MS (93) found only 17 out of 22 of the predicted sites. These differences could be due to the different experimental design and procedures used. Moreover, the composition diversity (oligomannose, complex, or hybrid-type), and frequency of these glycosylation events were dissimilar in both studies.

O-linked glycosylations were predicted at three previously proposed sites flanking the S1/S2 cleavage site (138). These events were not found in two recent mass spectrometry studies (93, 132). Thus, occurrence of these events could be very rare or affected by intra-host conditions. Interestingly, T323 and S325 in RBD were detected as O-glycosylated (93), which may be related to increasing affinity with the human receptor ACE2 (110, 139). N- and O-linked glycosylation events can influence not only their position but also the surrounding area under the glycan shield. In fact, both N- and O-linked glycosylations could be associated with masking epitopes or important amino acids, resulting in immune evasion (129, 140, 141). Further *in-vivo* studies should be performed to determine the real complexity and heterogeneity of these events.

Meanwhile, palmitoylation predicted sites in the cytoplasmic tail of the S protein (Table S8) have been reported in other Coronaviruses (34). The presence of these sites supports the importance of membrane-proximal cysteine-rich clusters in processes like the Spike-mediated cell fusion (27, 95, 142), infectivity (143), and viral assembly (144, 145).

Conservation of PTMs in amino acids can support the selection of candidate epitopes. In contrast, their emergence or disappearance resulting in better fitness should be considered important evolutive events and must be particularly monitored. In sum, the emergence/disappearance of alternative codons affecting PTMs in the evolutive course of the current pandemic is intriguing, and it has already been noticed in some SARS-CoV-2 genomes (73). Additional studies may unravel the impact of these events and could help in the development of strategies to control the infectious viral cycle.

In summary, our study provides updated HLA allele frequencies for South America, rectifying previously misrepresented alleles. This led to the identification of potential Class-I and -II epitopes in SARS-CoV-2 with high regional coverage. Some are supported by existing experimental evidence, while the rest represent novel candidates. These could represent targets for neutralizing antibodies or could be used for the development of vaccines and diagnostics tests, which needs to be further studied. Our study highlights the advantage of a regionally-focused design. Approaches based on the global population have the advantage of broad coverage. However, this may result in leaving aside the best regional candidates and reducing the regional population coverage, as shown in this study. Furthermore, incorrect HLA frequencies could result in misleading results and misrepresentation of certain populations. We hope our findings may promote regional efforts with potentially better specificity.

Additionally, the exuberant immune response against SARS-CoV-2 infections is related to disease severity (146, 147). COVID-19 has shown cases of minimal manifestations in people within the risk group as well as fatal response in people without apparent risk, thereby suggesting a genetic predisposition (148). In that sense, information about the HLA allele frequencies distribution in different populations may contribute to study the magnitude of the immune response and its severity.

Lack of knowledge of the HLA allele frequencies' distribution in South America also limits regional scientific studies in the field. This impacts the study of infectious and autoimmune diseases, cancer immunotherapy, and transplantations. Our results will serve as an immediate source of information for multiple ongoing studies based on HLA allele frequencies.

In conclusion, the candidate epitopes presented may help in the fight against SARS-CoV-2, providing valuable information for the development of peptide vaccines and diagnostic tests. And updated HLA allele frequencies will impact on the study of many human diseases. We hope this literature review may result in a better representation of South America in future immunogenetic studies.

## Data Availability Statement

All datasets generated for this study are included in the article/Supplementary Material.

## Author Contributions

DR and RC devised the research project. DR, AM, and OM-S performed the literature review. DR, MR, and RC selected and analyzed the viral genomes. DR, AM, and MR performed the epitope predictions. DR, AM, MR, and OM-S matched the predicted epitopes with previous experimental data and published predictions. AM and MR modeled the protein structures. AM, DR, and RC performed the prediction of selection pressures and post-translational modifications. AM and DR prepared the figures. All authors contributed to analyzing the results and manuscript preparation.

## Conflict of Interest

The authors declare that the research was conducted in the absence of any commercial or financial relationships that could be construed as a potential conflict of interest.

## Abbreviations

AFNDB: Allele Frequency Net Database
COVID-19: Coronavirus Disease 2019
CS: Coverage Score
GISAID: Global Initiative on Sharing All Influenza Data database
HLA: Human Leukocyte Antigen
IEDB: Immune Epitope Database and Analysis Resource
PDB: Protein Data Bank
PTM: Post-translational modifications
SARS-CoV-2: Severe Acute Respiratory Syndrome Coronavirus 2
WAF: Weighted Allele Frequency.
