# Supplementary material for: Identification of Novel Candidate Epitopes on SARS-CoV-2 Proteins for South America: A Review of HLA Frequencies by Country
Source: Front Immunol. 2020 Sep 3;11:2008. doi: 10.3389/fimmu.2020.02008 (PMC7494848; doi:10.3389/fimmu.2020.02008)
Supplement: Supplementary file 1 [file Data_Sheet_1.zip › Supplementary_Methods_final.pdf]

SUPPLEMENTARY METHODS

1. Selection of datasets and calculation of Weighted Allele Frequencies by country:

To calculate Weighted Allele Frequencies (WAFs) in the current and updated scenarios, we selected studies following the selection criteria described in the Methods section. As mentioned, some studies were not compatible, which led to few exceptions. For the sake of reproducibility, we are providing below the selection process in full detail. This is summarized in Table S3.

1.1 *Current Scenario, collecting datasets in the AFNDB:*

- Argentina:

  Applying the auxiliary rule for HLA class I, we obtained two datasets of Fernández-Viña *et al.*, 1997 (1) (Toba: N = 84 for A and N = 116 for B, Wichi: N = 44 for A and N = 46 for B). And two datasets of Marcos, 2000 (Toba: N = 86 for A and B, and Chiriguano: N = 54 for A and B), unpublished but available in the AFNDB. Nevertheless, we did not consider Chiriguano (N = 54) because most alleles where characterized at 2-digit resolution and just a 3 were characterized at 4-digit resolution. There were no datasets with 4-digit resolution for HLA-I C. For HLA class II, we did not need to apply the auxiliary rule. We included the studies of Cerna *et al.*, 1993 (N = 116) (2) and Marcos, 2000 (N = 365) for DPB1, DQA1, DQB1 and DRB1. We did not include the study of Motta *et al.*, 2007 (3) for DQB1 because it did not resolve most of the alleles at 4-digit resolution.

- Bolivia:

  For HLA-II we collected the datasets of Martinez-Laso *et al.*, 2006 (N = 80) (4) and Arnaiz-Villena *et al.*, 2005 (N = 102) (5), which report alleles of DRB1 with 4-digit resolution. There are no studies with 4-digit resolution for HLA class I. We are including the 2-digit resolution data of these two studies in Tables S4 and S5 just for illustrative purposes. These values were not used in any further analysis.

- Brazil:

  We applied the auxiliary rule for HLA-I, including the datasets of Donadi, 2002 (N = 108, available in the AFNDB) and Nunes *et al.*, 2016 (N = 144) (6) for the genes A, B and C. Also, the datasets of Middleton *et al.*, 2000 (N = 95 for A) (7), Williams *et al.*, 2001 (N = 95 for B) (8) and Lazaro *et al.*, 1999 (N = 60 for A and N = 58 for B) (9).

  For HLA class II, we applied the auxiliary rule for DPA1 and DPB1, including the datasets of Erlich, 2002 (N = 50, available in the AFNDB) for DPA1 and DPB1, and Cerna *et al.*,1993 (N = 74) (2) for DPB1. We did not need to apply the auxiliary rule for DQA1, DQB1 and DRB1. For these 3 genes, we included the studies of Castillo Lima Vendramini *et al.*, 2020 (N = 641)

(10), Maciag *et al.*, 2019 (N = 205, data taken from the AFNDB) (11) and Tsuneto *et al.*, 2003, which reports two datasets (First dataset: N=160 for these 3 genes; Second dataset: N = 235 for DQA1 and DRB1, N = 209 for DQB1) (12). Also, Temin *et al.*, 2002 (N = 103, available in the AFNDB) for both DQB1 and DRB1. And the datasets reported by Piovezan *et al.*, 2019 (N = 203) (13), Donadi, 2002 (N = 108) and Nunes *et al.*, 2016 (N = 144) (6) for DRB1 only.

- Chile:

We did not find studies with 100 or more individuals for HLA class I. By applying the auxiliary rule, we obtained the datasets of Arnaiz-Villena *et al.*, 2019 (N = 66 for A and B) (14) and Turner *et al.*, 2005 (N = 70), only available in the AFNDB. For HLA class II, we used the two studies mentioned above and the datasets reported by Pérez-Bravo *et al.*, 1999 (N = 124, for DQA1 and DQB1) (15) and Schäfer *et al.,* 2016 (N = 920, DRB1) (16).

- Colombia:

For HLA-I, using our inclusion criteria, we only obtained the study of Páez-Gutiérrez *et al.*, 2019 (N = 1463) (17) for A, B and C. For HLA-II, we applied the auxiliary rule for DPB1 and DQA1, selecting the datasets of Briceno *et al.*, 1996 (N = 54) for DPB1 (18) and Yunis *et al.*, 1994 (19) for DQA1. From this last study, as just the number of alleles in each dataset were provided, we are using N = 61.5 and N = 55.5 as approximation of the sample sizes. Of note, the frequencies reported for DPB1 and DQA1 are based on specific ethnic groups and may not be representative of the country diversity.

For genes DQB1 and DRB1, we only included the study of Páez-Gutiérrez *et al.*, 2019 (N = 1463, PCR-SBT) (17) and not the studies of Correa *et al.*, 2002 (N = 100, PCR-SSP) (20) and Arnaiz-Villena *et al.*, 2018 (N = 188, PCR-SSOP) (21) due to big differences in sample size and superior resolution technology (22,23).

- Ecuador:

By applying the auxiliary rule for HLA-I A, we included the study of Arnaiz-Villena *et al.*, 2018 (N = 63) (24). For HLA-I B, we only considered the integrated dataset of Galarza *et al.*, 2018 (N = 1101) (25) and not its subpopulations, which are also available individually on the AFNDB. We did not find studies of HLA-I C meeting our inclusion criteria.

For HLA-II, we applied the auxiliary rule for genes DPA1, DPB1, DQA1 and DQB1. For DPA1, we only found the dataset reported by Begovich *et al.*, 2001 (N = 58, Ecuadorian African) (26). For DPB1, we are using the datasets of Begovich *et al.*, 2001 (N = 58, Ecuadorian African) (26) and Trachtenberg *et al.*, 1995 (N = 183, Cayapa), available in the AFNDB). This last dataset combines the samples reported by Begovich *et al.*, 2001 (N = 83, Cayapa) (26) and Trachtenberg *et al.*, 1995 (N = 100, Cayapa) (27).

For both DQA1 and DQB1, we considered the datasets of Begovich *et al.*, 2000 (N = 58, Ecuadorian African) (28) and Trachtenberg *et al.*, 1995 (N = 183, Cayapa), available in the AFNDB. This last merges the datasets published in Begovich *et al.*, 2000 (N = 83, Cayapa)

(28) and Trachtenberg *et al.*, 1995 (N=100, Cayapa) (27). For DRB1, we only obtained the dataset of Trachtenberg *et al.*, 1995 (N = 183), available only in the AFNDB.

- Paraguay:

We did not find any eligible dataset for HLA-I. By applying the auxiliary rule for HLA class II, we only obtained the dataset of Tsuneto *et al.*, 2003 (N = 87) (12) for DQA1, DQB1 and DRB1. Importantly, these HLA allelic frequencies came from to the Aché ethnic group and may not be representative of the country's diversity.

- Peru:

For HLA-I A and B, we only considered the dataset of Arnaiz-Villena *et al.*, 2009 (N = 105) (29), which is reported twice in the AFNDB. However, this comes from an ethnic group (Uros) and may not be representative of the country's diversity. We did not find any other eligible study, neither for HLA-I A, B, C nor HLA-II DQA1, even applying the auxiliary rule. For illustrative purposes only, we are showing the HLA-I C alleles at 2-digit resolution reported by de Pablo *et al.*, 2000 (N = 148) (30). We are using the dataset of Tsuneto *et al.*, 2003 (N = 44, Quechua) (12) for HLA-II DQA1 only and not for DRB1, because many HLAs were typified just at 2-digit resolution for this gene.
We applied the auxiliary rule for HLA-II DQB1 and DRB1, selecting the studies of Arnaiz-Villena *et al.*, 2009 (N = 105) (29) and Moscoso *et al.*, 2006 (N = 83) (31) for both genes; and de Pablo *et al.*, 2000 (N = 148) (30) for DQB1 only.

- Venezuela:

For HLA class I, we only found datasets from the ethnic groups Yucpa and Bari: Layrisse *et al.*, 2001 (N = 73) (32) and Layrisse *et al.*, 2006 (N = 55, available in the AFNDB only), respectively. In the first dataset, the authors were not able to differentiate the alleles C*03:02 and C*03:04. We assigned this frequency to C*03:04 only, to match with the information in second dataset for the same allele. Worth mentioning, as both studies were done on ethnic groups, they may not be representative of the country's diversity. For HLA class II, we only used the study of Layrisse *et al.*, 2001 (N = 73) mentioned before. And we did not use the dataset of Layrisse, 2006 (N = 96, available in the AFNDB) for DRB1, because this study characterized most HLAs just at 2-digit resolution and only two alleles at 4-digit resolution.

- Uruguay, Guyana, French Guiana, and Suriname: No reports in the AFNDB.

1.2 *Updated Scenario, adding datasets from the literature review:*

A literature review was performed to collect additional datasets from scientific articles for the Updated Scenario. We used the following search command in PubMed:

("HLA-A Antigens"[MeSH Terms] OR "HLA-B Antigens"[MeSH Terms] OR "HLA-C Antigens"[MeSH Terms] OR "HLA-DP Antigens"[MeSH Terms] OR "HLA-DQ Antigens"[MeSH Terms] OR "HLA-DRB1 Chains"[MeSH Terms] OR "HLA Antigens"[MeSH Terms] OR "HLA"[All Fields]) AND ("1990/01/01"[Date - Publication] : "2020/04/10"[Date - Publication]) AND ("Peru" OR "Bolivia" OR "Argentina" OR "Uruguay" OR "Brazil" OR "Chile" OR "Ecuador" OR "Paraguay" OR "Venezuela" OR "Colombia" OR "Suriname" OR "Guyana" OR "French Guiana" OR "Amerindian")

This command can be used directly in PubMed's website. However, we used the python script search_pubmed_entrez (33), which produces a table with the articles information organized in columns. The results were manually verified.

Then, datasets were selected following our inclusion criteria (see Figure 1). Some of the articles collected (11,13,15,34–49) investigated correlation of HLA alleles with certain diseases. Therefore, as they found risk/protective alleles in the study group, we decided to use the data of the control group only.

The procedure below describes how the datasets collected by country were integrated together with the datasets already selected for the current scenario. This is summarized in Table S3.

- Argentina:

For HLA class I, we only considered the study of Hurley *et al.*, 2017 (N = 1472 for A, B and C, typified by PCR-SBT) (50) due to its technological (22,23) and numerical differences versus the other eligible datasets described in the current scenario: Fernández-Viña *et al.*, 1997 (PCR-SSOP) (1) and Marcos *et al.*, 2000 (PCR-SSOP). For HLA-II, we are including the studies of Motta *et al.*, 2014 (N = 119, DQB1) (45), Fainboim *et al.*, 1994 (N = 103, DQB1) (43) and Pando *et al.*, 1999 (DQA1, DQB1 and DRB1) (51). In this last article, frequencies were not shown for all the alleles typified. However, we found the complete dataset in the first author's doctoral thesis (44).

- Bolivia:

For HLA-II, we are adding a dataset reported by Cervantes *et al.*, 2003 (N = 105) (46) for DQB1 and DRB1. As we found no studies for HLA-I with 4-digit resolution, we are including the 2-digit resolution data of Cervantes *et al.*, 2003 in Tables S4 and S5, just for illustrative purposes. These values were not used in any further analysis.

- Brazil:

For HLA class I, we are only using the study of Halagan *et al.*, 2018 (N = 4.18M for A and B, N = 92,569 for C, PCR-SBT) (52) and not the datasets of Donadi 2002 (available in the AFNDB), Nunes *et al.*, 2016 (6), Braun-Prado *et al.*, 2000 (53), Fabreti-Olivera *et al.*, 2014

(54), Castro *et al.*, 2019 (55) and Werneck *et al.*, 2016 (34), due to notorious differences in sample size and technology (22,23).

For HLA-II DPA1 and DPB1, we are only using the studies of Castro *et al.*, 2019 (N = 108 for DPA1 and DPB1, PCR-SBT) (55) and Werneck *et al.*, 2016 (N = 133 for DPB1, PCR-SBT) (34). For DQA1, we are including the datasets of Franco-Brochado *et al.*, 2016 (N = 1312, PCR-SSOP) (47), Cardozo *et al.*, 2014 (N = 173, PCR-SSOP) (48), Castro *et al.*, 2019 (N = 108, PCR-SBT) (55), and Sotomaior *et al.*, 1998 (N = 108, PCR-SSOP) (56). Sotomoaior *et al.*, 1998 (56) were not able to differentiate DQA1*01:01 and DQA1*01:04, reporting a single frequency value for both alleles. We assigned this value to DQA1*01:01 due to its high abundance in the other studies considered. We did not include the study of Shimokawa *et al.*, 2016 (N = 103, PCR-SSP) (49), because the genotypification technique used has lower resolution. We did not consider the study of Reis *et al.*, 2018 (N = 641 for DQA1 and DQB1, PCR-SSOP) (57) since it reports the same control group (although in less detail) of the study of Castillo Lima Vendramini *et al.*, 2020 (already included in the current scenario).

For DQB1 and DRB1, we only used the dataset of Halagan *et al.*, 2018 (N = 193,053 for DQB1, N = 4.18M for DRB1, PCR-SBT) (52). Due to clear differences in sample size and technology (22,23), we did not consider the studies neither the studies in the current scenario, nor the studies of Cangussu *et al.*, 2011 (N = 103, PCR-SSP) (58), da Costa Lima Caniatti *et al.*, 2017 (N = 143, PCR-SSOP) (59), Donadi *et al.*, 2000 (N = 119, PCR-SSOP) (60), Fabreti-Oliveira *et al.*, 2014 (N = 551, PCR-SSOP) (54), Fernandes *et al.*, 2002 (N = 181, PCR-SSOP) (61), Freitas *et al.*, 2004 (N = 166, PCR-SSP) (62), Braun-Prado *et al.*, 2018 (N = 149 to N = 158, PCR-SSOP) (53), Gil *et al.*, 2017 (N = 297, PCR-SSOP) (63), Louzada-Junior *et al.*, 2008 (N = 156, PCR-SSOP) (64), Souza de Lima *et al.*, 2016 (N = 101, PCR-SBT) (65), Usnayo *et al.*, 2011 (N = 215, PCR-SSOP) (66), and Kay *et al.*, 2019 (N = 215, PCR-SBT) (67) and Maciel *et al.*, 2001 (N = 166, PCR-SSP) (68).

- <u>Chile:</u>

In addition to the datasets mentioned in the current scenario, we found three studies meeting our inclusion criteria: Castro-Santos *et al.,* 2020 (N =160 for HLA-I A, B, C and N = 510 for HLA II DRB1) (69), Rey *et al.,* 2013 (N = 104 for HLA-I A and B, HLA-II DQB1 and DRB1) (70), and Perez-Bravo *et al.,* 1995 (N = 74, for HLA-II DQA1 and DQB1) (35). For genes DQA1 and DQB1, we did not include the articles of Díaz *et al.,* 2003 (N = 125) (71) and Santos *et al.,* 2000 (N = 255) (72) because these studies were just focused on detecting the presence/absence of few specific alleles.

- <u>Colombia:</u>

For HLA-II, by applying the auxiliary rule for DPB1, we are including two additional datasets, each of N = 50, reported by Briceno *et al.*, 1996 (18). For DQB1 and DRB1, we are adding the study of Del Río-Ospina *et al.*, 2019 (N = 274) (73).

- Ecuador:

No additional dataset was found for HLA-I. We applied the auxiliary rule for HLA-II, adding the study of Begovich *et al.*, 2001 (N = 83, Cayapa) for DPA1 (26). Additionally, for DQA1 and DQB1 we are including the datasets reported by De Angelis *et al.*, 2011 (N = 79) (36) and Iorio *et al.*, 2014 (N = 52) (74).

- Paraguay:

The only study that met our selection criteria is Benitez *et al.*, 2011 (N = 40, for HLA-I A, B and HLA-II DRB1) (75).

- Peru:

For HLA-I, we included the study of Olvera *et al.*, 2015 (N = 468, Lima) (37), which screened alleles of HLA-I A, B and C in 246 HIV-positive and 222 HIV-negative individuals by PCR-SBT. They found statistically significant differences in the frequency of 4 alleles, for which we used the frequencies of the control group: 9.86% for B*40:02, 1.76% for B*35:43, 1.76% for A*23:01 and 0% for B*57:03. We did not include the dataset of Arnaiz-Villena *et al.*, 2009 (N = 105) (29) for HLA-I due to technological discrepancy (22,23).

- Uruguay:

For HLA-I, we have not found datasets with 4-digit resolution. We are showing the largest study with 2-digit resolution, Alvarez *et al.*, 2006 (N = 959), in Tables S4 and S5 for illustrative purposes only (76). These values were not used in any further analysis.
For HLA-II, we only obtained the dataset of Mimbacas *et al.*, 2003 (38), reporting the allelic frequencies of DQB1 in 80 control patients.

- Venezuela:

For genes DPA1, DPB1, DQA1 and DQB1, by applying the auxiliary rule, we are including the datasets reported by Balducci-Silano *et al.*, 1995 (N = 147 for DPA1 and DPB1) (39), Rivera-Pirela *et al.*, 2016 (N = 48 for DPA1 and DPB1) (77) and Gendzekhadze *et al.*, 2004 (N = 44 for DPB1) (78), Makhatadze *et al.*, 1997 (N = 164 for DQA1 and N = 165 for DQB1) (79), Dao *et al.*, 2005 (N = 79 for DQB1) (42) and Sáenz-Cantele *et al.*, 2007 (N = 101 for DQB1) (40) in addition to the dataset of Layrisse *et al.*, 2001 (N = 73) (32), already considered in the current scenario. We are assigning the frequency reported by Sáenz-Cantele *et al.*, 2007 for DQB1*02 to DQB1*02:01, since is it the only variant of DQB1*02 reported in the other datasets for this country.

For HLA-II DRB1, it was not necessary to apply the auxiliary rule. We are only considering the datasets of Makhatadze *et al.*, 1997 (N = 160) (79), Sáenz-Cantele *et al.*, 2007 (N = 101) (40) and Fortes *et al.*, 2007 (N = 111) (41).

- Guyana, French Guiana, and Suriname: No eligible datasets were found.

1.3 *Weighted Allele Frequencies by country:*

WAFs by country were calculated for both the current and updated scenarios as described in the Methods section, using the data considered in each scenario as described above (see Table 3). However, not all the datasets selected by country reported identical set of alleles. A small number of alleles had frequencies reported in just some but not all the studies, which could be either an actual reflection of the population studied or caused by study particularities (small sample size not representative enough of the total population, study on ethnic or isolated groups, among other possibilities). In these cases, the weighted average calculation considers the value of zero for allele frequencies not reported in a study. This caused some weighted average frequencies to drop below the 5% cut-off. Therefore, as we are uncertain if this WAF values are trustworthy, we are also reporting the WAF values calculated ignoring the missing values. These are shown enclosed in brackets in Tables S5 and S6.

Additionally, few datasets were not entirely characterized at 4-digit resolution, reporting the frequencies of some alleles with 2-digit resolution only. For those alleles affected, we calculated the WAF using only the information available at 4-digit resolution. Therefore, in these cases we have alleles with lower sample sizes than others of the same gene in the same country. These numbers are shown enclosed in curly brackets in Tables S5 and S6.

2. References:

1. Fernández-Viña MA, Lázaro AM, Marcos CY, Haas EJ. Dissimilar evolution of B-locus versus A-locus and class II loci of the HLA region in South American Indian tribes. *Tissue Antigens* (1997) **50**:233–250. doi:10.1111/j.1399-0039.1997.tb02867.x PMID - 9331946

2. Cerna M, Falco M, Friedman H, Raimondi E, Maccagno A, Fernandez-Viña M, Stastny P. Differences in HLA class II alleles of isolated South American Indian populations from Brazil and Argentina. *Hum Immunol* (1993) **37**:213–220. doi:10.1016/0198-8859(93)90504-t PMID - 8300406

3. Motta PMF, Cech N, Fontan C, Giménez MF, Lodeiro N, Marinic K, Molinari ML, Sotelo MG, Sorrentino Ah. [Role of HLA-DR and HLA-DQ alleles in multibacillary leprosy and paucibacillary leprosy in the province of Chaco (Argentina)]. *Enferm Infecc Microbiol Clin* (2007) **25**:627–631. doi:10.1157/13112938 PMID - 18053473

4. Martinez-Laso J, Siles N, Moscoso J, Zamora J, Serrano-Vela JI, R-A-Cachafeiro JI, Castro MJ, Serrano-Rios M, Arnaiz-Villena A. Origin of Bolivian Quechua Amerindians: their relationship with other American Indians and Asians according to HLA genes. *Eur J Med Genet* (2006) **49**:169–185. doi:10.1016/j.ejmg.2005.04.005 PMID - 16530714

5. Arnaiz-Villena A, Siles N, Moscoso J, Zamora J, Serrano-Vela J, Gomez-Casado E, Castro M, Martinez-Laso J. Origin of Aymaras from Bolivia and their relationship with other Amerindians according to HLA genes. *Tissue Antigens* (2005) **65**:379–390. doi:10.1111/j.1399-0039.2005.00356.x PMID - 15787722

6. Nunes K, Piovezan B, Torres MA, Pontes GN, Kimura L, Carnavalli JE, Netto RCM, Moraes ME, Meyer D. Population variation of HLA genes in rural communities in Brazil, the Quilombos from the Vale do Ribeira, São Paulo – Brazil. *Hum Immunol* (2016) **77**:447–448. doi:10.1016/j.humimm.2016.04.007 PMID - 27060779

7. Middleton D, Williams F, Meenagh A, Daar AS, Gorodezky C, Hammond M, Nascimento E, Briceno I, Perez MP. Analysis of the distribution of HLA-A alleles in populations from five continents. *Hum Immunol* (2000) **61**:1048–1052. doi:10.1016/S0198-8859(00)00178-6

8. Williams F, Meenagh A, Darke C, Acosta A, Daar AS, Gorodezky C, Hammond M, Nascimento E, Middleton D. Analysis of the distribution of HLA-B alleles in populations from five continents. *Hum Immunol* (2001) **62**:645–650. doi:10.1016/S0198-8859(01)00247-6

9. Lázaro AM, Moraes M, Marcos CY, Moraes J, Fernández-Viña MA, Stastny P. Evolution of HLA-class I compared to HLA-class II polymorphism in Terena, a South-American Indian tribe. *Hum Immunol* (1999) **60**:1138–1149. doi:10.1016/s0198-8859(99)00092-0 PMID - 10600013

10. Castillo Lima Vendramini E, de Lima Neto QA, Guimarães Reis P, Moliterno RA, de Souza VH, Sell AM, Laguila Visentainer JE. HLA-A, -B, -DRB1, -DQA1, and -DQB1 genotyping of 641 individuals from southern Brazil. *Hum Immunol* (2020) **81**:8–9. doi:10.1016/j.humimm.2019.12.001

11. Maciag PC, Schlecht NF, Souza PS, Franco EL, Villa LL, Petzl-Erler ML. Major histocompatibility complex class II polymorphisms and risk of cervical cancer and human papillomavirus infection in Brazilian women. *Cancer Epidemiol Biomarkers Prev* (2000) **9**:1183–1191.

12. Tsuneto L, Probst C, Hutz M, Salzano F, Rodriguez-Delfin L, Zago M, Hill K, Hurtado A, Ribeiro-dos-Santos A, Petzl-Erler M. HLA class II diversity in seven Amerindian populations. Clues about the origins of the Aché. *Tissue Antigens* (2003) **62**:512–526. doi:10.1046/j.1399-0039.2003.00139.x PMID - 14617035

13. Piovezan BZ, Petzl-Erler ML. Both qualitative and quantitative genetic variation of MHC class II molecules may influence susceptibility to autoimmune diseases: The case of endemic pemphigus foliaceus. *Hum Immunol* (2013) **74**:1134–1140.

doi:10.1016/j.humimm.2013.06.008 PMID - 23777927

14. Arnaiz-Villena A, Juarez I, Lopez-Nares A, Palacio-Grüber J, Vaquero C, Callado A, Sevillano A, Rey D, Martin-Villa JM. Frequencies and significance of HLA genes in Amerindians from Chile Cañete Mapuche. *Hum Immunol* (2019) **80**:419–420. doi:10.1016/j.humimm.2019.04.015 PMID - 31101374

15. Pérez-Bravo F, Araya M, Mondragón A, Ríos G, Alarcón T, Roessler JL, Santos JL. Genetic differences in HLA-DQA1* and DQB1* allelic distributions between celiac and control children in Santiago, Chile. *Hum Immunol* (1999) **60**:262–267. doi:10.1016/S0198-8859(98)00119-0

16. Schäfer C, Sauter J, Riethmüller T, Kashi ZM, Schmidt AH, Barriga FJ. HLA-A, -B, -DRB1 allele and haplotype frequencies of 920 cord blood units from Central Chile. *Hum Immunol* (2016) **77**:622–623. doi:10.1016/j.humimm.2016.05.020 PMID - 27233642

17. Páez-Gutiérrez IA, Hernández-Mejía DG, Vanegas D, Camacho-Rodríguez B, Perdomo-Arciniegas AM. HLA-A, -B, -C, -DRB1 and -DQB1 allele and haplotype frequencies of 1463 umbilical cord blood units typed in high resolution from Bogotá, Colombia. *Hum Immunol* (2019) **80**:425–426. doi:10.1016/j.humimm.2019.03.006

18. Briceno I, Gomez A, Bernal JE, Papiha SS. HLA-DPB 1 POLYMORPHISM IN SEVEN SOUTH AMERICAN INDIAN TRIBES IN COLOMBIA. *Int J Immunogenet* (1996) **23**:235–240. doi:10.1111/j.1744-313x.1996.tb00118.x PMID - 8803536

19. Yunis JJ, Ossa H, Salazar M, Delgado MB, Deulofeut R, Hoz A, Bing DH, Ramos O, Yunis EJ, Yunis EJ. Major histocompatibility complex class II alleles and haplotypes and blood groups of four Amerindian tribes of Northern Colombia. *Hum Immunol* (1994) **41**:248–258. doi:10.1016/0198-8859(94)90043-4 PMID - 7883592

20. Correa P, Whitworth W, Kuffner T, McNicholl J, Anaya J. HLA-DR and DQB1 gene polymorphism in the North-western Colombian population. *Tissue Antigens* (2002) **59**:436–439. doi:10.1034/j.1399-0039.2002.590515.x PMID - 12144632

21. Arnaiz-Villena A, Bayona B, Palacio-Gruber J, Hernández E, Muñiz E, Campos C, Juarez I, Gomez-Casado E, Martín-Villa JM, Silvera C. HLA genes in Barranquilla (North Colombia): Searching for cryptic Amerindian genes. *Hum Immunol* (2018) **79**:3–4. doi:10.1016/j.humimm.2017.11.003 PMID - 29129648

22. Smith AG, Pereira S, Jaramillo A, Stoll ST, Khan FM, Berka N, Mostafa AA, Pando MJ, Usenko CY, Bettinotti MP, et al. Comparison of sequence-specific oligonucleotide probe vs next generation sequencing for HLA-A, B, C, DRB1, DRB3/B4/B5, DQA1, DQB1, DPA1, and DPB1 typing: Toward single-pass high-resolution HLA typing in support of solid organ and hematopoietic cell transp. *HLA* (2019) **94**:296–306. doi:10.1111/tan.13619 PMID - 31237117

23. Spínola H, Bruges-Armas J, Brehm A. Discrepancies in HLA typing by PCR-SSOP and SBT techniques: a case study. *Hum Biol* (2007) **79**:537–543. doi:10.1353/hub.2008.0001

PMID - 18478969

24. Arnaiz-Villena A, Palacio-Gruber J, Salamanca Me, Juárez I, Campos C, Nieto J, Muñiz E, Martin-Villa JM. HLA-G, -A haplotypes in Amerindians (Ecuador): HLA-G*01:05N World distribution. *Hum Immunol* (2018) **79**:89–90. doi:10.1016/j.humimm.2017.12.002 PMID - 29217368

25. Galarza JM, Barquera R, Álvarez AMT, Zaragoza DIH, Sevilla GP, Tamayo A, Pérez M, Dávila D, Birnberg L, Alonzo VA, et al. Genetic diversity of the HLA system in human populations from the Sierra (Andean), Oriente (Amazonian) and Costa (Coastal) regions of Ecuador. *Hum Immunol* (2018) **79**:639–650. doi:10.1016/j.humimm.2018.06.004 PMID - 29908213

26. Begovich A, Moonsamy P, Mack S, Barcellos L, Steiner L, Grams S, Suraj-Baker V, Hollenbach J, Trachtenberg E, Louie L, et al. Genetic variability and linkage disequilibrium within the HLA-DP region: analysis of 15 different populations. *Tissue Antigens* (2001) **57**:424–439. doi:10.1034/j.1399-0039.2001.057005424.x PMID - 11556967

27. Trachtenberg EA, Erlich HA, DeStefano GF. HLA class II linkage disequilibrium and haplotype evolution in the Cayapa Indians of Ecuador. *Am J Hum Genet* (1995) **57**:415–424.

28. Begovich AB, Klitz W, Steiner LL, Grams S, Suraj-Baker V, Hollenbach J, Trachtenberg E, Louie L, Zimmerman PA, Hill AVS, et al. *HLA-DQ haplotypes in 15 different populations*. , ed. S. Japan (2000). Available at: https://www.springer.com/gp/book/9784431702764

29. Arnaiz-Villena A, Gonzalez-Alcos V, Serrano-Vela JI, Reguera R, Barbolla L, Parga-Lozano C, Gómez-Prieto P, Abd-El-Fatah-Khalil S, Moscoso J. HLA genes in Uros from Titikaka Lake, Peru: Origin and relationship with other Amerindians and worldwide populations. *Int J Immunogenet* (2009) **36**:159–167. doi:10.1111/j.1744-313X.2009.00841.x

30. de Pablo R, Beraún Y, Nieto A, Calzada J, Rementería M, Sanz L, López-Nevot M, Martín J. HLA class I and class II allele distribution in the Peruvian population. *Tissue Antigens* (2000) **56**:507–514. doi:10.1034/j.1399-0039.2000.560604.x PMID - 11169240

31. Moscoso J, Seclen S, Serrano-Vela JI, Villena A, Martinez-Laso J, Zamora J, Moreno A, IRA-Cachafeiro J, Arnaiz-Villena A. HLA genes in Lamas Peruvian–Amazonian Amerindians. *Mol Immunol* (2006) **43**:1881–1889. doi:10.1016/j.molimm.2005.10.013 PMID - 16337001

32. Layrisse Z, Guedez Y, Domínguez E, Paz N, Montagnani S, Matos M, Herrera F, Ogando V, Balbas O, Rodríguez-Larralde A. Extended HLA haplotypes in a carib amerindian population: the Yucpa of the Perija Range. *Hum Immunol* (2001) **62**:992–1000. doi:10.1016/s0198-8859(01)00297-x PMID - 11543901

33. Requena D. Search_pubmed_entrez (First release). (2020). *Zenodo* (2020) doi:10.5281/zenodo.3733158

34. Werneck LC, Lorenzoni PJ, Arndt RC, Kay CSK, Scola RH. The immunogenetics of multiple sclerosis. The frequency of HLA-alleles class 1 and 2 is lower in Southern Brazil than in the European population. *Arq Neuropsiquiatr* (2016) **74**:607–616. doi:10.1590/0004-282x20160100 PMID - 27556370

35. Perez-Bravo F, Serrano-Rios M, Gutierrez-Lopez M, Calvillan M, Garcia de los Rios M, Kocova M, Dorman J, Trucco M. Genetic analysis of HLA DRB1, DQA1 and DQB1 alleles and susceptibility to IDDM in Chilean subjects. *Diabetologia* (1995) **38**:378–379. doi:10.1007/BF00400647

36. De Angelis F, Garzoli A, Battistini A, Iorio A, Stefano GFD. Genetic response to an environmental pathogenic agent: HLA-DQ and onchocerciasis in northwestern Ecuador. *Tissue Antigens* (2011) **79**:123–129. doi:10.1111/j.1399-0039.2011.01811.x PMID - 22117902

37. Olvera A, Pérez-Álvarez S, Ibarrondo J, Ganoza C, Lama JR, Lucchetti A, Cate S, Hildebrand W, Bernard N, Gomez L, et al. The HLA-C*04: 01/KIR2DS4 gene combination and human leukocyte antigen alleles with high population frequency drive rate of HIV disease progression. *AIDS* (2015) **29**:507–517. doi:10.1097/qad.0000000000000574 PMID - 25715101

38. Mimbacas A, Pérez-Bravo F, Hidalgo PC, Javiel G, Pisciottano C, Grignola R, Jorge AM, Gallino JP, Gasagoite J, Cardoso H. Association between diabetes type 1 and DQB1 alleles in a case-control study conducted in Montevideo, Uruguay. PMID - 12917799. *Genet Mol Res* (2003) **2**:29–35.

39. Balducci-Silano PL, Layrisse ZE. HLA-DP and Susceptibility to Insulin-Dependent Diabetes Mellitus in an Ethnically Mixed Population. Associations with Other HLA-Alleles. *J Autoimmun* (1995) **8**:425–437. doi:10.1006/jaut.1995.0034 PMID - 7576003

40. Sáenz-Cantele AM, Fernández-Mestre M, Montagnani S, Calebotta A, Balbas O, Layrisse Z. HLA-DRB1*0402 haplotypes without DQB1*0302 in Venezuelan patients with pemphigus vulgaris. *Tissue Antigens* (2007) **69**:318–325. doi:10.1111/j.1399-0039.2007.00826.x PMID - 17389015

41. Fortes M, Machado I V, Gil G, Fernández-Mestre M, Dagher L, León R V, Bianco NE, Tassinari P. Genetic contribution of major histocompatibility complex class II region to type 1 autoimmune hepatitis susceptibility in Venezuela. *Liver Int* (2007) **27**:1409–1416. doi:10.1111/j.1478-3231.2007.01581.x PMID - 17927716

42. Dao DD, Sierra-Torres CH, Robazetti SC, Gomez Mn, König R, Lema C, Lester LJ, Au WW, Tyring SK. HLA-DQB1 and cervical cancer in Venezuelan women. *Gynecol Oncol* (2005) **96**:349–354. doi:10.1016/j.ygyno.2004.10.002 PMID - 15661220

43. Fainboim L, Marcos Y, Pando M, Capucchio M, Reyes GB, Galoppo C, Badía I,

Remondino G, Ciocca M, Ramonet M, et al. Chronic active autoimmune hepatitis in children Strong association with a particular HLA-DR6 (DRB1∗1301) haplotype. *Hum Immunol* (1994) **41**:146–150. doi:10.1016/0198-8859(94)90008-6 PMID - 7860360

44. Pando M. Estudio molecular de la distribución de alelos HLA-DR-DQ en poblaciones normales argentinas y en pacientes con hepatitis crónica autoinmune. (1998) Available at: https://bibliotecadigital.exactas.uba.ar/download/tesis/tesis_n3093_Pando.pdf

45. Motta PMF, López MA, Marinic K, Picón SO, Stafuza MG, Sorrentino AH de. [High Frequency of DQ8 in the Celiac Population of Chaco Province, Argentina]. *Acta Gastroenterol Latinoam* (2014) **44**:16–21.

46. Cervantes J, Lema C, Hurtado L V, Andrade R, Gomez LH, Torrico L, Zegarra L, Quiroga G, Asturizaga D, Dulon A, et al. HLA-DRB1*1602 allele is positively associated with HPV cervical infection in Bolivian Andean women. *Hum Immunol* (2003) **64**:890–895. doi:10.1016/s0198-8859(03)00163-0 PMID - 12941545

47. Franco Brochado MJ, Nascimento DF, Deghaide NHS, Donadi EA, Roselino AM. Data on HLA class I/II profile in Brazilian pemphigus patients. *Data Br* (2016) **8**:364–374. doi:10.1016/j.dib.2016.05.077 PMID - 27331116

48. Cardozo DM, Moliterno RA, Sell AM, Guelsin GAS, Beltrame LM, Clementino SL, Reis PG, Alves H V, Mazini PS, Visentainer JEL. Evidence of HLA-DQB1 Contribution to Susceptibility of Dengue Serotype 3 in Dengue Patients in Southern Brazil. *J Trop Med* (2014) **2014**:968262. doi:10.1155/2014/968262 PMID - 24817893

49. Shimokawa PT, Targa LS, Yamamoto L, Rodrigues JC, Kanunfre KA, Okay TS. HLA-DQA1/B1 alleles as putative susceptibility markers in congenital toxoplasmosis. *Virulence* (2016) **7**:456–464. doi:10.1080/21505594.2016.1150401 PMID - 26856406

50. Hurley CK, Hou L, Lazaro A, Gerfen J, Enriquez E, Galarza P, Cardozo MBR, Halagan M, Maiers M, Behm D, et al. Next generation sequencing characterizes the extent of HLA diversity in an Argentinian registry population. *HLA* (2018) **91**:175–186. doi:10.1111/tan.13210 PMID - 29327506

51. Pando M, Larriba J, Fernandez GC, Fainboim H, Ciocca M, Ramonet M, Badia I, Daruich J, Findor J, Tanno H, et al. Pediatric and adult forms of type I autoimmune hepatitis in Argentina: Evidence for differential genetic predisposition. *Hepatology* (1999) **30**:1374–1380. doi:10.1002/hep.510300611

52. Halagan M, Oliveira DC, Maiers M, Fabreti-Oliveira RA, Moraes MEH, Visentainer JEL, Pereira NF, Romero M, Cardoso JF, Porto LC. The distribution of HLA haplotypes in the ethnic groups that make up the Brazilian Bone Marrow Volunteer Donor Registry (REDOME). *Immunogenetics* (2018) **70**:511–522. doi:10.1007/s00251-018-1059-1

53. Braun-Prado K, Mion A V, Pereira NF, Culpi L, Petzl-Erler M. HLA class I polymorphism, as characterised by PCR-SSOP, in a Brazilian exogamic population. *Tissue Antigens* (2000) **56**:417–427. doi:10.1034/j.1399-0039.2000.560504.x PMID -

11144289

54. Fabreti-Oliveira RA, Nascimento E, Fonseca CG, Santos MA. The heterogeneous HLA genetic composition of the Brazilian population and its relevance to the optimization of hematopoietic stem cell donor recruitment. *Tissue Antigens* (2014) **84**:187–197. doi:10.1111/tan.12352 PMID - 24724906

55. Castro M, Issler HC, Gelmini GF, Miranda Blm, Calonga-Solís V, Schmidt AH, Stein A, Bicalho M, Petzl-Erler ML, Augusto DG. High-resolution characterization of 12 classical and non-classical HLA loci in Southern Brazilians. *HLA* (2019) **93**:80–88. doi:10.1111/tan.13488 PMID - 30740929

56. Sotomaior VS, Faucz FR, Boldt AB, Petzl-Erler ML. HLA-DQA1 and HLA-DQB1 alleles and haplotypes in two Brazilian Indian tribes: evidence of conservative evolution of HLA-DQ. *Hum Biol* (1998) **70**:789–797.

57. Reis PG, Ambrosio-Albuquerque EP, Fabreti-Oliveira RA, Moliterno RA, Souza Vh, Sell AM, Visentainer JEL. HLA-A, -B, -DRB1, -DQA1, and -DQB1 profile in a population from southern Brazil. *HLA* (2018) **92**:298–303. doi:10.1111/tan.13368 PMID - 30225991

58. Cangussu LOF, Teixeira R, Campos EF, Rampim GF, Mingoti SA, Martins-Filho OA, Gerbase-DeLima M. HLA Class II Alleles and Chronic Hepatitis C Virus Infection. *Scand J Immunol* (2011) **74**:282–287. doi:10.1111/j.1365-3083.2011.02568.x PMID - 21535077

59. Caniatti Mc, Borelli SD, Guilherme ALF, Tsuneto LT. Association between HLA genes and dust mite sensitivity in a Brazilian population. *Hum Immunol* (2017) **78**:88–94. doi:10.1016/j.humimm.2016.10.014 PMID - 27780790

60. Donadi EA, Smith AG, Louzada-Júnior P, Voltarelli J, Nepom GT. HLA class I and class II profiles of patients presenting with Sydenham's chorea. *J Neurol* (2000) **247**:122–128. doi:10.1007/s004150050027 PMID - 10751115

61. FERNANDES APM, LOUZADA-JÚNIOR P, FOSS MC, DONADI EA. HLA-DRB1, DQB1, and DQA1 Allele Profile in Brazilian Patients with Type 1 Diabetes Mellitus. *Ann N Y Acad Sci* (2002) **958**:305–308. doi:10.1111/j.1749-6632.2002.tb02992.x PMID - 12021129

62. Freitas M V, Silva Lm, Deghaide NH, Donadi EA. Is HLA class II susceptibility to primary antiphospholipid syndrome different from susceptibility to secondary antiphospholipid syndrome? *Lupus* (2004) **13**:125–131. doi:10.1191/0961203304lu520oa PMID - 14995006

63. Gil JM, Weber R, Rosales CB, Rodrigues H, Sennes LU, Kalil J, Chagury A, Miziara ID. Study of the association between human leukocyte antigens (HLA) and pemphigus vulgaris in Brazilian patients. *Int J Dermatol* (2017) **56**:557–562. doi:10.1111/ijd.13577 PMID - 28197992

64. Louzada-Júnior P, Freitas M, Oliveira R, Deghaide N, Conde R, Bertolo M, Donadi E. A

majority of Brazilian patients with rheumatoid arthritis HLA-DRB1 alleles carry both the HLA-DRB1 shared epitope and anti-citrunillated peptide antibodies. *Brazilian J Med Biol Res* (2008) **41**:493–499. doi:10.1590/s0100-879x2008005000021 PMID - 18560674

65. De Lima DS, Ogusku MM, Dos Santos MP, De Melo Silva CM, De Almeida VA, Antunes IA, Boechat AL, Ramasawmy R, Sadahiro A. Alleles of HLA-DRB1∗04 associated with pulmonary tuberculosis in Amazon Brazilian population. *PLoS One* (2016) **11**: doi:10.1371/journal.pone.0147543

66. Usnayo MJG, Andrade LEC, Alarcon RT, Oliveira JC, Silva GMF, Bendet I, Burlingame R, Porto LC, Pinheiro G. Study of the frequency of HLA-DRB1 alleles in Brazilian patients with rheumatoid arthritis. *Rev Bras Reumatol* (2011) **51**:474–483. doi:10.1590/s0482-50042011000500007 PMID - 21952999

67. Kay CSK, Scola RH, Arndt RC, Lorenzoni PJ, Werneck LC. HLA-alleles class I and II associated with genetic susceptibility to neuromyelitis optica in Brazilian patients. *Arq Neuropsiquiatr* (2019) **77**:239–247. doi:10.1590/0004-282x20190031 PMID - 31090804

68. Maciel LM, Rodrigues SS, Dibbern RS, Navarro PA, Donadi EA. Association of the HLA-DRB1*0301 and HLA-DQA1*0501 alleles with Graves' disease in a population representing the gene contribution from several ethnic backgrounds. *Thyroid* (2001) **11**:31–35. doi:10.1089/10507250150500630 PMID - 11272094

69. Castro-Santos P, Olloquequi J, Díaz-Peña R. HLA-A, B, C and DRB1 alleles in a Chilean population from Talca. *HLA* (2020) **95**:200–203. doi:10.1111/tan.13775 PMID - 31846232

70. Rey D, Parga-Lozano C, Moscoso J, Areces C, Enriquez-de-Salamanca M, Fernández-Honrado M, Abd-El-Fatah-Khalil S, Alonso-Rubio J, Arnaiz-Villena A. HLA genetic profile of Mapuche (Araucanian) Amerindians from Chile. *Mol Biol Rep* (2013) **40**:4257–4267. doi:10.1007/s11033-013-2509-3 PMID - 23666052

71. Díaz N, Méndez M, Pérez-Bravo F, Carrasco E, Santos J. Incidence rate of type 1 diabetes in Santiago (Chile) by HLA-DQA1 and DQB1 genotypes. *Eur J Epidemiol* (2003) **18**:787–792. doi:10.1023/a:1025345804789 PMID - 12974555

72. Santos J. Associations between HLA-DQB1 high-risk alleles and type I diabetes do not depend on cytomegalovirus antibody status at onset: A case-parent study conducted in Chile. *Immunol Cell Biol* (2000) **78**:259–263. doi:10.1046/j.1440-1711.2000.00911.x PMID - 10849114

73. Río-Ospina LD, Camargo M, León SCS, Robayo-Calderón KL, Garzón-Ospina D, Patarroyo ME, Patarroyo MA. Using next-generation sequencing for characterising HLA-DRB1 and DQB1 loci in a cohort of Colombian women. *HLA* (2019) **94**:425–434. doi:10.1111/tan.13672 PMID - 31441245

74. Iorio A, Angelis FD, Garzoli A, Battistini A, Stefano GFD. HLA-DQA1 and HLA-DQB1 genes in Tsachilas Indians from Ecuador: new insights in population analysis by Human

Leukocyte Antigens. *Int J Immunogenet* (2014) **41**:222–230. doi:10.1111/iji.12116 PMID - 24775353

75. Benitez O, Busson M, Charron D, Loiseau P. HLA polymorphism in a Guarani-Indian population from Paraguay and its usefulness for the Hispano-Indian admixture study in Paraguay. *Int J Immunogenet* (2011) **38**:7–11. doi:10.1111/j.1744-313x.2010.00959.x PMID - 20670332

76. Alvarez I, Bengochea M, Toledo R, Carretto E, Hidalgo PC. HLA class I antigen and HLA-A, -B, and -C haplotype frequencies in Uruguayans. *Hum Biol* (2006) **78**:513–525. doi:10.1353/hub.2006.0051

77. Rivera-Pirela SE, Echeverría M, Salcedo P, Márquez G, Carrillo Z, Parra Y, Cipriani AM, Núñez JR, De Mon MÁ. HLA DRB1*, DQB1*, DPA1*, and DPB1* and their association with the pathogenesis of leukemia in the population of Venezuela. *Rev Alerg Mex* (2016) **63**:237–251. doi:10.29262/ram.v63i3.147

78. Gendzekhadze K, Herrera F, Montagnani S, Balbas O, Witter K, Albert E, Layrisse Z. HLA-DP polymorphism in Venezuelan Amerindians. *Hum Immunol* (2004) **65**:1483–1488. doi:10.1016/j.humimm.2004.07.238

79. Makhatadze NJ, Franco MT, Layrisse Z. HLA Class I and Class II Allele and Haplotype Distribution in the Venezuelan Population. *Hum Immunol* (1997) **55**:53–58. doi:10.1016/s0198-8859(97)00088-8 PMID - 9328790
