# Supplementary material for: Identification of Novel Candidate Epitopes on SARS-CoV-2 Proteins for South America: A Review of HLA Frequencies by Country
Source: Front Immunol. 2020 Sep 3;11:2008. doi: 10.3389/fimmu.2020.02008 (PMC7494848; doi:10.3389/fimmu.2020.02008)
Supplement: Supplementary file 1 [file Data_Sheet_1.zip › Tables_1_and_2.docx]

**TABLE 1.** Best HLA-I candidate epitopes for South America in SARS-CoV-2 proteins.

| **Best HLA-I candidate epitopes for South America** | | | | | | **N**° **of alleles with WAF ≥ 0.05 covered by country** | | | | | | | | | **Coverage Score** | **Reference** |
| --- | --- | --- | --- | --- | --- | --- | --- | --- | --- | --- | --- | --- | --- | --- | --- | --- |
| **Protein** | **Start** | **End** | **Peptide** | **Experiment** | **IEDB ID** | **ARG** | **BOL** | **BRA** | **CHI** | **COL** | **ECU** | **PAR** | **PER** | **VEN** |  |  |
| S | 1060 | 1068 | VVFLHVTYV | LTM, ML | 71663 | 3 | - | 5 | 3 | 6 | 1 | 2 | 3 | 9 | 2.146 | Ahmed *et al.* (65) |
|  | 894 | 902 | LQIPFAMQM | ML | 38855 | 1 | - | 4 | 2 | 5 | 0 | 6 | 1 | 11 | 2.043 |  |
| ORF6 | 3 | 11 | HLVDFQVTI | ML | 24313 | 3 | - | 6 | 4 | 6 | 1 | 3 | 3 | 12 | 2.520 | New |
| NSP3 | 950 | 958 | VMYMGTLSY |  | 5477, 70040 | 6 | - | 7 | 5 | 7 | 1 | 5 | 2 | 8 | 2.801 |  |
| NSP5 | 219 | 227 | F**L**NRFTTTL |  | 16786 | 6 | - | 9 | 6 | 8 | 1 | 3 | 4 | 13 | 3.224 |  |
| NSP6 | 86 | 94 | MPASWVMRI |  | 42260, 42261 | 4 | - | 6 | 5 | 6 | 0 | 3 | 3 | 9 | 2.292 |  |
| NSP8 | 47 | 55 | SEFDRDAAM |  | 57419 | 3 | - | 2 | 3 | 4 | 1 | 5 | 0 | 7 | 1.832 |  |
| NSP12 | 877 | 885 | YADVFHLYL | ML | 14969 | 6 | - | 11 | 7 | 8 | 1 | 3 | 5 | 10 | 3.346 | New |
|  | 123 | 131 | TMADLVYAL |  | 65176, 65177 | 3 | - | 5 | 4 | 6 | 1 | 4 | 4 | 13 | 2.756 |  |
|  | 898 | 906 | HMLDMYSVM |  | 24342 | 3 | - | 5 | 3 | 5 | 1 | 4 | 4 | 12 | 2.591 |  |
| NSP13 | 355 | 363 | YVFCTVNAL | ML | 76266 | 6 | - | 9 | 6 | 8 | 1 | 3 | 5 | 13 | 3.335 | New |
|  | 291 | 299 | FAIGLA**L**YY |  | 23758 | 6 | - | 9 | 7 | 8 | 1 | 3 | 3 | 6 | 2.838 |  |
| NSP14 | 494 | 502 | YLDAYNMMI | ML | 74593 | 5 | - | 9 | 6 | 8 | 1 | 1 | 4 | 11 | 2.823 | New |
|  | 500 | 508 | MMISAGFSL |  | 42128 | 2 | - | 5 | 4 | 6 | 1 | 4 | 3 | 13 | 2.590 |  |
| S | 869 | 877 | MIAQYTSAL | - | - | 8 | - | 10 | 8 | 9 | 1 | 5 | 6 | 15 | 4.127 | New |
|  | 691 | 699 | SIIAYTMSL |  |  | 6 | - | 8 | 6 | 9 | 1 | 5 | 5 | 16 | 3.739 |  |
|  | 269 | 277 | YLQPRTFLL |  |  | 6 | - | 8 | 6 | 8 | 2 | 3 | 6 | 14 | 3.646 |  |
| NSP2 | 420 | 428 | YITGGVVQL | - | - | 5 | - | 8 | 6 | 9 | 1 | 5 | 4 | 12 | 3.382 | New |
|  | 265 | 273 | GLNDNLLEI |  |  | 2 | - | 4 | 2 | 5 | 1 | 1 | 3 | 7 | 1.705 |  |
| NSP3 | 1776 | 1784 | YVNTFSSTF | - | - | 6 | - | 8 | 7 | 9 | 1 | 4 | 4 | 10 | 3.276 | New |
|  | 1452 | 1460 | YLNSTNVTI |  |  | 5 | - | 7 | 6 | 9 | 1 | 3 | 4 | 14 | 3.180 |  |
|  | 816 | 824 | YYHTTD**P**SF |  |  | 6 | - | 9 | 8 | 9 | 1 | 2 | 5 | 8 | 3.148 |  |
| NSP4 | 25 | 33 | YLITPVHVM | - | - | 6 | - | 7 | 6 | 9 | 1 | 6 | 5 | 14 | 3.721 | New |
|  | 309 | 317 | **G**EYSHVVAF |  |  | 3 | - | 3 | 3 | 5 | 1 | 6 | 1 | 9 | 2.270 |  |
| NSP12 | 442 | 450 | FAQDGNA**A**I | - | - | 8 | - | 12 | 8 | 10 | 1 | 4 | 6 | 12 | 4.013 | New |
|  | 281 | 289 | KLFDRYFKY |  |  | 4 | - | 4 | 4 | 4 | 1 | 5 | 2 | 5 | 2.169 |  |
| NSP16 | 103 | 111 | FVSDADSTL | - | - | 6 | - | 11 | 7 | 9 | 1 | 3 | 4 | 13 | 3.437 | New |

**TABLE 2.** Best HLA-II candidate epitopes for South America in SARS-CoV-2 proteins.

| **Best HLA-II candidate epitopes for South America** | | | | | | | **N**° **of alleles with WAF ≥ 0.05 covered by country** | | | | | | | | | **Coverage Score** | **Reference** |
| --- | --- | --- | --- | --- | --- | --- | --- | --- | --- | --- | --- | --- | --- | --- | --- | --- | --- |
| **Protein** | **Start** | **End** | **Peptide** | **Region** | **Experiment** | **IEDB ID** | **ARG** | **BOL** | **BRA** | **CHI** | **COL** | **ECU** | **PAR** | **PER** | **VEN** |  |  |
| S | 1013 | 1027 | IRAAEIRASANLAAT | CH | LTM | 100428 | 5 | 5 | 5 | 4 | 5 | 7 | 4 | 7 | 4 | 8.148 | New |
|  | 1014 | 1028 | RAAEIRASANLAATK |  |  | 100428 | 6 | 5 | 7 | 4 | 5 | 7 | 5 | 7 | 5 | 9.000 |  |
|  | 1152 | 1166 | LDKYFKNHTSPDVDL | HR2 | ML | 35205 | 4 | 4 | 4 | 3 | 4 | 5 | 5 | 6 | 3 | 6.760 | Ahmed *et al.* (65) |
|  | 1153 | 1167 | DKYFKNHTSPDVDLG |  |  | 9006 | 4 | 4 | 4 | 3 | 4 | 5 | 5 | 6 | 3 | 6.760 |  |
| S | 61 | 75 | NVTWFHAIHVSGTNG | NTD | - | - | 4 | 4 | 4 | 3 | 4 | 5 | 4 | 4 | 4 | 6.474 | New |
|  | 114 | 128 | TQSLLIVNNATNVVI |  |  |  | 4 | 4 | 5 | 3 | 4 | 6 | 5 | 6 | 3 | 7.045 |  |
|  | 115 | 129 | QSLLIVNNATNVVIK |  |  |  | 5 | 4 | 6 | 4 | 5 | 5 | 5 | 5 | 4 | 7.719 |  |
|  | 116 | 130 | SLLIVNNATNVVIKV |  |  |  | 4 | 3 | 5 | 3 | 4 | 4 | 5 | 4 | 4 | 6.474 |  |
|  | 206 | 220 | KHTPINLVRDLPQGF |  |  |  | 4 | 3 | 4 | 3 | 4 | 5 | 3 | 5 | 3 | 6.017 |  |
|  | 207 | 221 | HTPINLVRDLPQGFS |  |  |  | 5 | 4 | 5 | 3 | 4 | 6 | 5 | 6 | 4 | 7.412 |  |
|  | 208 | 222 | TPINLVRDLPQGFSA |  |  |  | 4 | 4 | 3 | 3 | 4 | 5 | 2 | 6 | 3 | 6.017 |  |
|  | 216 | 230 | LPQGFSALEPLVDLP |  |  |  | 3 | 4 | 3 | 3 | 3 | 5 | 5 | 6 | 4 | 6.450 |  |
|  | 217 | 231 | PQGFSALEPLVDLPI |  |  |  | 3 | 4 | 3 | 3 | 3 | 5 | 5 | 6 | 4 | 6.450 |  |
|  | 308 | 322 | VEKGIYQTSNFRVQP | RBD | - | - | 4 | 5 | 5 | 3 | 4 | 7 | 5 | 7 | 3 | 7.531 |  |
|  | 309 | 323 | EKGIYQTSNFRVQPT |  |  |  | 5 | 5 | 6 | 4 | 5 | 7 | 5 | 7 | 4 | 8.490 |  |
|  | 313 | 327 | YQTSNFRVQPTESIV |  |  |  | 3 | 5 | 3 | 3 | 3 | 6 | 5 | 6 | 3 | 6.593 |  |
|  | 314 | 328 | QTSNFRVQPTESIVR |  |  |  | 6 | 5 | 7 | 4 | 5 | 6 | 5 | 6 | 5 | 8.714 |  |
|  | 315 | 329 | TSNFRVQPTESIVRF |  |  |  | 6 | 5 | 7 | 4 | 5 | 7 | 5 | 7 | 5 | 9.000 |  |
|  | 316 | 330 | SNFRVQPTESIVRFP |  |  |  | 3 | 4 | 5 | 3 | 3 | 5 | 5 | 5 | 4 | 6.593 |  |
|  | 430 | 444 | TGCVIAWNSNNLDSK |  |  |  | 4 | 4 | 6 | 3 | 4 | 6 | 5 | 6 | 4 | 7.388 |  |
|  | 689 | 703 | SQSIIAYTMSLGAEN | - | - | - | 3 | 5 | 3 | 3 | 4 | 7 | 4 | 7 | 3 | 6.879 |  |
|  | 690 | 704 | QSIIAYTMSLGAENS |  |  |  | 4 | 5 | 4 | 3 | 4 | 7 | 5 | 7 | 3 | 7.388 |  |
|  | 785 | 799 | VKQIYKTPPIKDFGG |  |  |  | 3 | 5 | 2 | 3 | 3 | 5 | 4 | 6 | 3 | 6.107 |  |
|  | 801 | 815 | NFSQILPDPSKPSKR | FP | - | - | 5 | 4 | 5 | 3 | 4 | 6 | 4 | 6 | 4 | 7.212 |  |
|  | 802 | 816 | FSQILPDPSKPSKRS |  |  |  | 5 | 4 | 5 | 3 | 4 | 6 | 5 | 6 | 4 | 7.412 |  |
|  | 1059 | 1073 | GVVFLHVTYVPAQEK | BH | - | - | 3 | 5 | 3 | 3 | 3 | 7 | 5 | 7 | 4 | 7.079 |  |
|  | 1060 | 1074 | VVFLHVTYVPAQEKN |  |  |  | 3 | 4 | 2 | 3 | 3 | 5 | 4 | 6 | 4 | 6.107 |  |
|  | 1098 | 1112 | NGTHWFVTQRNFYEP | SD3 | - | - | 4 | 4 | 4 | 3 | 3 | 5 | 5 | 5 | 4 | 6.617 |  |
|  | 1099 | 1113 | GTHWFVTQRNFYEPQ |  |  |  | 4 | 4 | 4 | 3 | 3 | 6 | 5 | 6 | 4 | 6.902 |  |
|  | 1110 | 1124 | YEPQIITTDNTFVS**G** |  |  |  | 4 | 4 | 4 | 3 | 4 | 5 | 5 | 5 | 4 | 6.817 |  |
|  | 1111 | 1125 | EPQIITTDNTFVS**G**N |  |  |  | 4 | 4 | 6 | 3 | 4 | 6 | 5 | 6 | 4 | 7.388 |  |
|  | 1126 | 1140 | CDVVIGIVNNTVYDP | - | - | - | 4 | 3 | 5 | 3 | 4 | 4 | 5 | 4 | 4 | 6.474 |  |
| M | 7 | 21 | TITVEELKKLLEQWN | Virion Surface | - | - | 2 | 2 | 2 | 1 | 1 | 3 | 3 | 3 | 2 | 3.326 | New |
| E | 1 | 15 | MYSFVSEETGTLIVN | Virion Surface | - | - | 0 | 2 | 1 | 1 | 1 | 2 | 0 | 2 | 1 | 1.764 | New |
