# Supplementary figures and images for: Identification of Novel Candidate Epitopes on SARS-CoV-2 Proteins for South America: A Review of HLA Frequencies by Country
Source: Front Immunol. 2020 Sep 3;11:2008. doi: 10.3389/fimmu.2020.02008 (PMC7494848; doi:10.3389/fimmu.2020.02008)

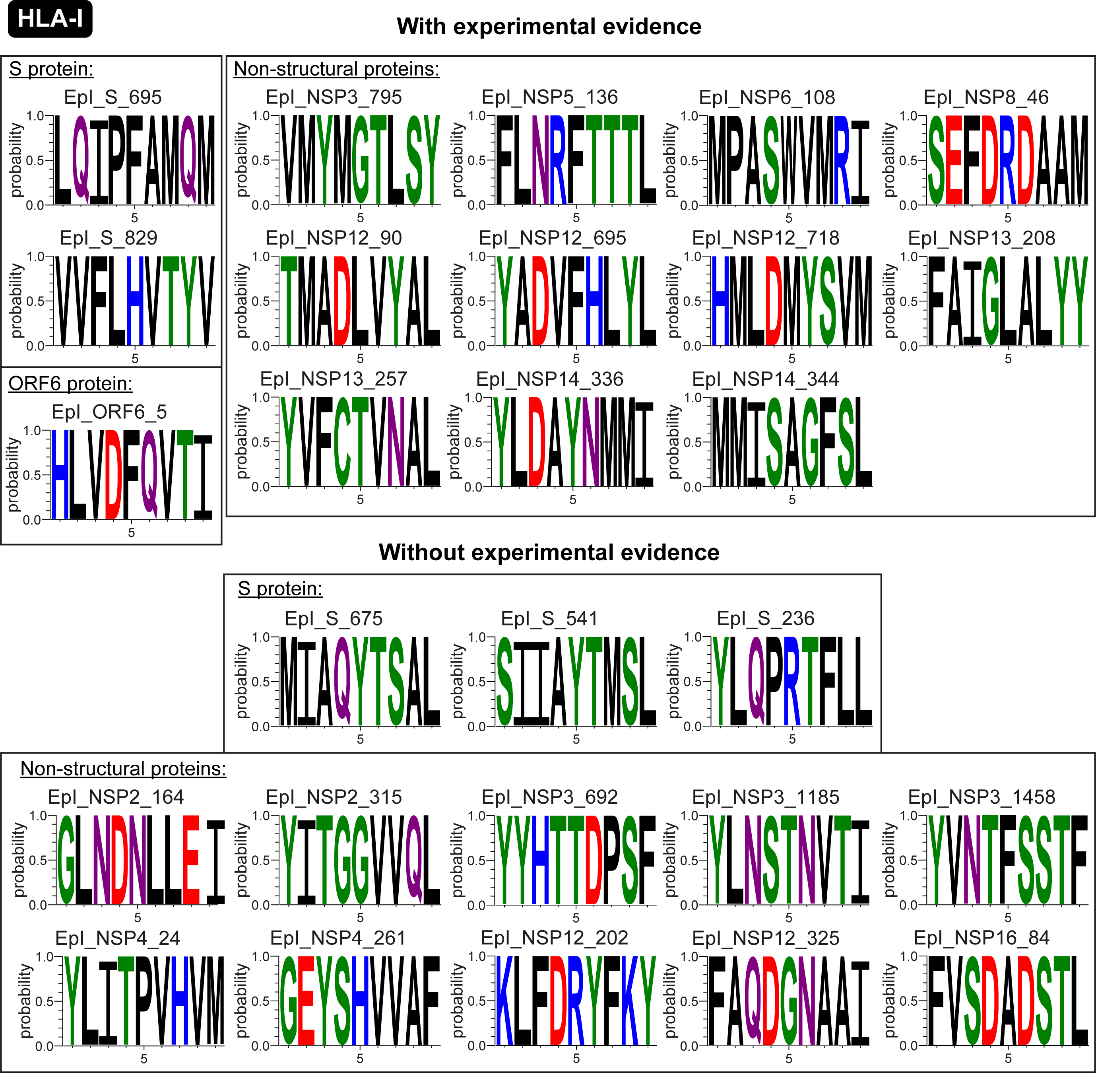

Supplement: Supplementary file 1 [file Data_Sheet_1.zip › Figure_S1_final.tif]

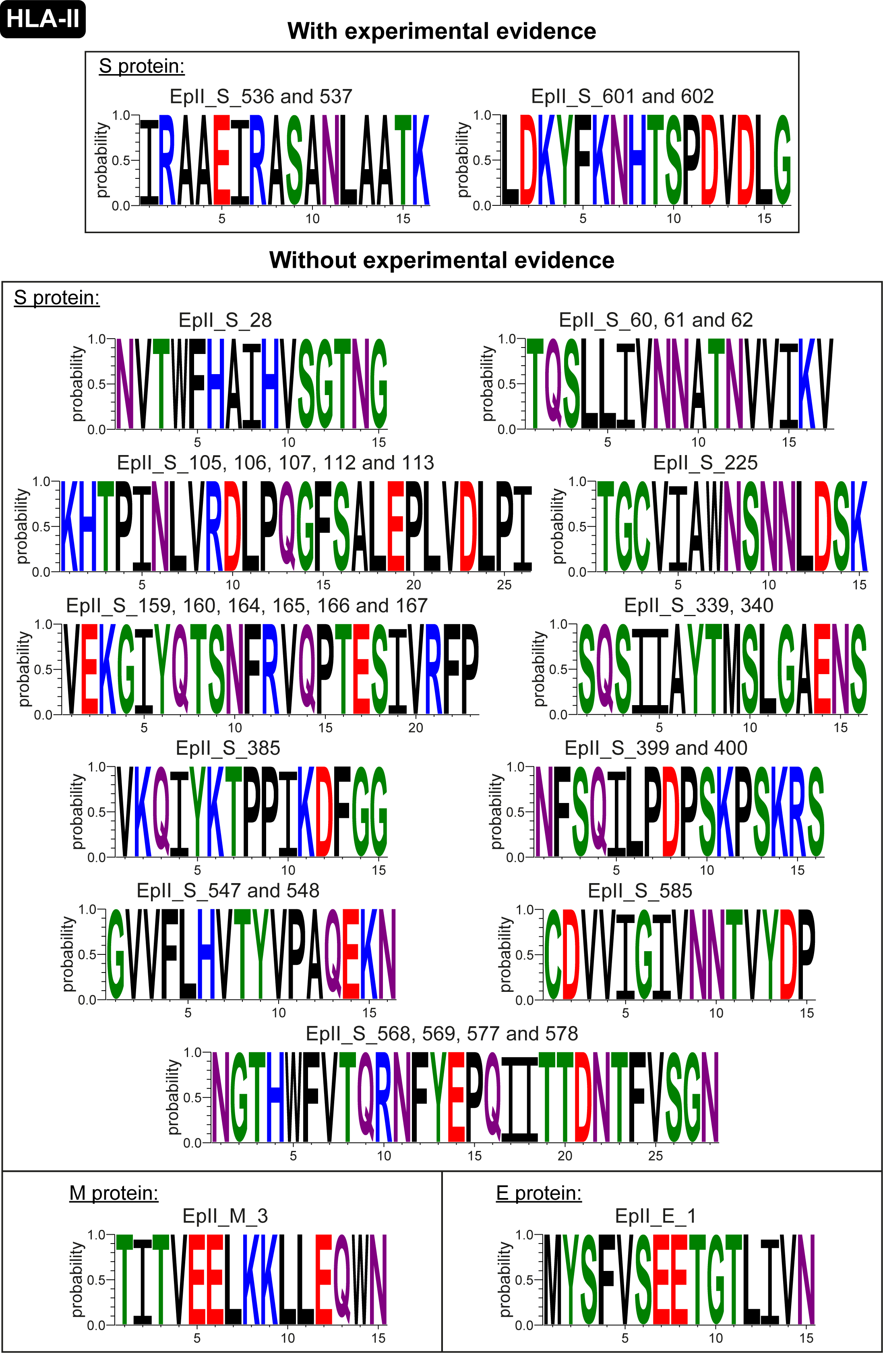

Supplement: Supplementary file 1 [file Data_Sheet_1.zip › Figure_S2_final.tif]
